# Inhibition of ATM or ATR in combination with hypo-fractionated radiotherapy leads to a different immunophenotype on transcript and protein level in HNSCC

**DOI:** 10.3389/fonc.2024.1460150

**Published:** 2024-10-01

**Authors:** Julia Meidenbauer, Matthias Wachter, Sebastian R. Schulz, Nada Mostafa, Lilli Zülch, Benjamin Frey, Rainer Fietkau, Udo S. Gaipl, Tina Jost

**Affiliations:** ^1^ Translational Radiobiology, Department of Radiation Oncology, Uniklinikum Erlangen, Friedrich-Alexander-Universität Erlangen-Nürnberg, Erlangen, Germany; ^2^ Department of Radiation Oncology, Uniklinikum Erlangen, Friedrich-Alexander-Universität Erlangen-Nürnberg, Erlangen, Germany; ^3^ Comprehensive Cancer Center Erlangen-Europäische Metropolregion Nürnberg (EMN), Uniklinikum Erlangen, Friedrich-Alexander-Universität Erlangen-Nürnberg, Erlangen, Germany; ^4^ Division of Molecular Immunology, Internal Medicine III, University Hospital Erlangen, Nikolaus-Fiebiger Center, Friedrich-Alexander-University Erlangen-Nürnberg, Erlangen, Germany; ^5^ Deutsches Zentrum Immuntherapie, Uniklinikum Erlangen, Erlangen, Germany; ^6^ FAU Profile Center Immunomedicine Friedrich-Alexander-Universität Erlangen-Nürnberg, Erlangen, Germany

**Keywords:** HNSCC, DNA damage repair, kinase inhibitors, immunomodulation, ATM inhibition, ATR inhibition

## Abstract

**Background:**

The treatment of head and neck tumors remains a challenge due to their reduced radiosensitivity. Small molecule kinase inhibitors (smKI) that inhibit the DNA damage response, may increase the radiosensitivity of tumor cells. However, little is known about how the immunophenotype of the tumor cells is modulated thereby. Therefore, we investigated whether the combination of ATM or ATR inhibitors with hypo-fractionated radiotherapy (RT) has a different impact on the expression of immune checkpoint markers (extrinsic), the release of cytokines or the transcriptome (intrinsic) of head and neck squamous cell carcinoma (HNSCC) cells.

**Methods:**

The toxic and immunogenic effects of the smKI AZD0156 (ATMi) and VE-822 (ATRi) in combination with a hypo-fractionated scheme of 2x5Gy RT on HPV-negative (HSC4, Cal-33) and HPV-positive (UM-SCC-47, UD-SCC-2) HNSCC cell lines were analyzed as follows: cell death (necrosis, apoptosis; detected by AnxV/PI), expression of immunostimulatory (ICOS-L, OX40-L, TNFSFR9, CD70) and immunosuppressive (PD-L1, PD-L2, HVEM) checkpoint marker using flow cytometry; the release of cytokines using multiplex ELISA and the gene expression of Cal-33 on mRNA level 48 h post-RT.

**Results:**

Cell death was mainly induced by the combination of RT with both inhibitors, but stronger with ATRi. Further, the immune phenotype of cancer cells, not dying from combination therapy itself, is altered predominantly by RT+ATRi in an immune-stimulatory manner by the up-regulation of ICOS-L. However, the analysis of secreted cytokines after treatment of HNSCC cell lines revealed an ambivalent influence of both inhibitors, as we observed the intensified secretion of IL-6 and IL-8 after RT+ATRi. These findings were confirmed by RNAseq analysis and further the stronger immune-suppressive character of RT+ATMi was enlightened. We detected the down-regulation of a central protein of cytoplasmatic sensing pathways of nucleic acids, RIG-1, and found one immune-suppressive target, EDIL3, strongly up-regulated by RT+ATMi.

**Conclusion:**

Independent of a restrictive toxicity, the combination of RT + either ATMi or ATRi leads to comprehensive and immune-modulating alterations in HNSCC. This includes pro-inflammatory signaling induced by RT + ATRi but also anti-inflammatory signals. These findings were confirmed by RNAseq analysis, which further highlighted the immune-suppressive nature of RT + ATMi.

## 1 Introduction

Head and neck squamous cell carcinoma (HNSCC) represent a heterogenous group of malignancies that originate from the epithelium in the larynx, pharynx and the oral cavity. Tobacco and alcohol consumption are the main risk factors for the development of HNSCC in the oral cavity and the larynx. Infection with human papillomavirus (HPV), particularly HPV-16, is increasingly associated with pharyngeal occurrences (1, 2). Notably, HPV-positive and HPV-negative cancers differ not only in terms of etiology and risk factors but also exhibit genetic distinctions and divergent survival rates (3, 4). Conventional treatment of HNSCC involves a multimodal concept incorporating surgical resection, radiotherapy (RT) and chemotherapy, often leading to impairment in speech and swallowing together with a range of other harmful side effects (5–7). Interestingly, HPV-positive HNSCC demonstrates superior overall survival and progression-free survival rates (3), partly due to heightened intrinsic sensitivity to radiation (4, 8). This intrinsic radiosensitivity in this case is promoted by the viral proteins E6 and E7. These proteins do not promote tumorigenesis by inducing cell immortality or migration solely, but interfere with the cell cycle and more important with p53-signaling (9). The p53 protein is degraded by E6, leading to the loss of cell cycle control meaning the tumor is in fact not able to stop the cell cycle adequality. However, a sufficient cell cycle stop is necessary for DNA damage repair, a consequence of radiotherapy-induced DNA damage. This results in a higher radiosensitivity of HPV-associated tumors compared to HPV-independent HNSCC. As a result, current efforts are directed towards identifying agents that can improve the compatibility and effectiveness of HNSCC treatment, with a focus on enhancing HPV-negative cancer treatment. One emerging approach in cancer treatment including HNSCC involves the use of small molecule kinase inhibitors (smKI) targeting Ataxia telangiectasia mutated (ATM) or Ataxia telangiectasia mutated and Rad3-related (ATR) kinases (10, 11). Both proteins belong to the phosphoinositide 3-kinase (PI3K) family and play a critical role in DNA double-strand break (DSB) repair (12).

Cancer cells are characterized by genomic instability, attributed in part to the inactivation of proteins involved in DNA damage repair (DDR) (12). The same mechanism is evident in the case of HPV infection, where the expression of oncogenes E6 and E7 results in the avoidance of apoptosis and inhibition of growth suppressor activities through the degradation of p53 and the retinoblastoma protein, respectively (13, 14). Cancer cells use this ability to circumvent cell cycle checkpoints while concurrently becoming dependent on crucial proteins for cell survival. As a result, cancer therapies that cause DNA damage often encounter relapse due to elevated levels of DDR proteins. Subsequently, targeting proteins crucial for DSB repair represents an opportunity to sensitize cancer cells to DNA-damaging treatments such as irradiation (12).

Two promising drugs in this context are AZD0156 and VE-822. AZD0156 is a highly potent and selective inhibitor of ATM that has been already shown to work as a radiosensitizer *in vitro* and *in vivo* and is currently being examined in phase I studies (NCT02588105) (15–17). VE 822, an ATR inhibitor also known as Berzosertib, significantly enhances radiosensitivity in several cancers such as esophageal cancer, colorectal cancer and melanoma and is currently being evaluated in phase I and II studies (18–20). We recently already showed that both AZD0156 and VE-822 synergistically inhibit tumor growth in HNSCC when combined with irradiation (21, 22).

However, only little is currently known how combined treatments of ATM or ATR inhibition in combination with RT affect the immunogenicity of HNSCC tumor cells. Recent findings indicate that HPV-positive HNSCC exhibits heightened immunogenicity compared to HPV-negative cancer, with a greater activation of immune signaling pathways and a higher number of tumor-infiltrating lymphocytes (23). In alignment with this observation, we recently found that RT induces an upregulation of the immunostimulatory ICOS-L only on HPV-positive HNSCC (24). The interaction between ICOS and ICOS-L is known to promote T cell activation and differentiation, underscoring its potential in the development of cancer immunotherapies (25).

We hypothesized that the combination of smKI with RT, in addition to enhancing the effects of irradiation, influences the immunogenicity of HNSCC in dependence of the HPV status. We expected the combined treatment to alter the expression of surface immune checkpoints molecules on the HNSCC cells as well as the secretion of pro- and anti-inflammatory cytokines by the cancer cells. This might result in the activation of CD8+ T cells and NK cells, thereby accelerating the anti-tumor response. To test our hypothesis, we treated two HPV-positive and two HPV-negative cells lines with RT, smKI or a combination of both. Subsequently, we analyzed tumor cell death forms, surface expression of immune checkpoint molecules, cytokine secretion and mRNA expression profiles of the treated HNSCC cells.

## 2 Materials and methods

### 2.1 Cell lines and cell culture

Two HPV-negative HSC4 (RRID: CVCL_1289), Cal-33 (RRID: CVCL_1108) and two HPV-positive UM-SCC-47 (RRID: CVCL_7759), UD-SCC-2 (RRID: CVCL_E325) HNSCC cell lines were generously provided by Dr. Thorsten Rieckmann at the University Medical Center Hamburg-Eppendorf. The cell lines were cultured in basic cell culture medium (DMEM, Pan-Biotech GmbH, Aidenbach, Germany) supplemented with 10% fetal bovine serum (FBS, Sigma-Aldrich, St. Louis, MO, USA) and 1% Penicillin-Streptomycin (PenStrep, Gibco, Waltham, MA, USA). Passage of the cell lines was performed twice a week using 0.5% Trypsin solution (Gibco Life Technologies, Carlsbad, CA, USA).

### 2.2 Treatments and irradiation scheme

**Figure 1:**
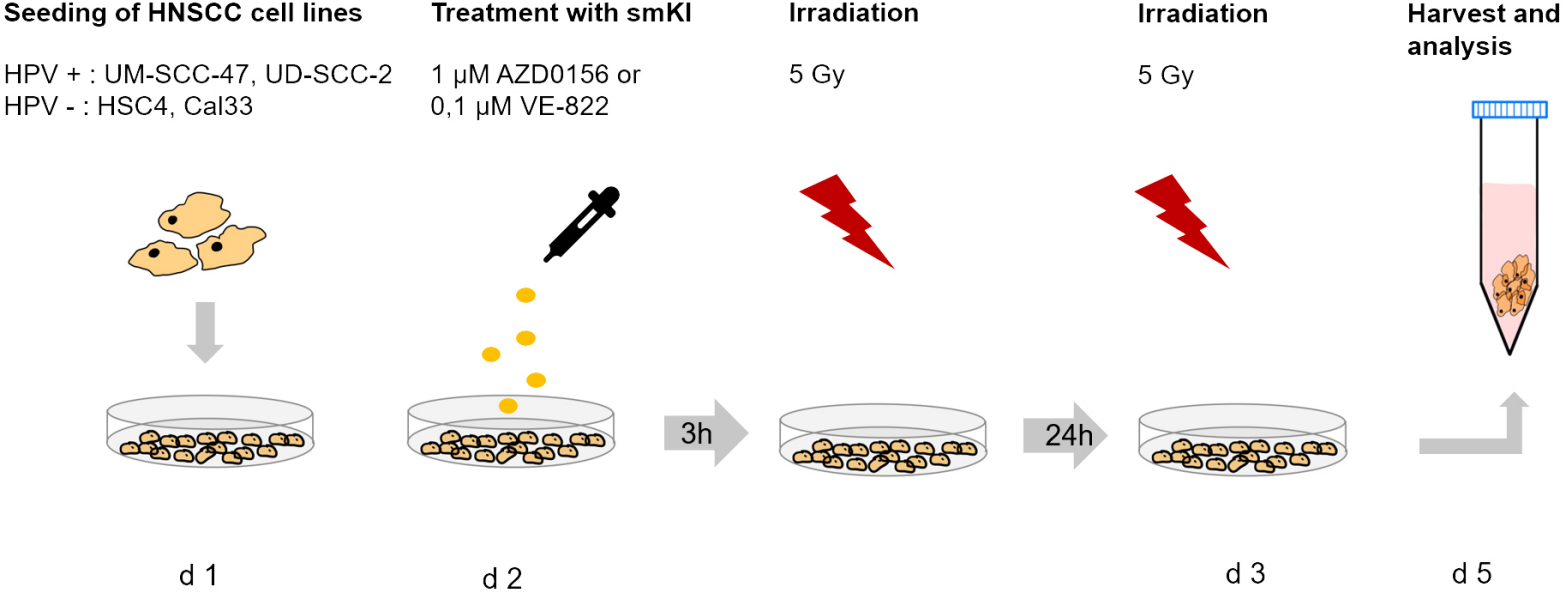
Treatment scheme of HNSCC cell lines. HPV-negative (HSC4, Cal-33) and HPV-positive (UM-SCC-47, UD-SCC-2) HNSCC cells were seeded on day 1 and treated with 1µM AZD0156 or 0,1 µM VE-822 24 hours later. Irradiation with 5 Gy was performed 3 hours after treatment with smKI and repeated 24 hours later. Cells were harvested and analyzed 24 hours after the last treatment.

Cells were exposed to the ATM inhibitor AZD0156 (Selleckchem, Houston, TX, USA) and the ATR inhibitor VE-822 (Selleckchem, Houston, TX, USA), both dissolved in dimethyl sulfoxide (DMSO, Roth, Karlsruhe, Germany) and stored at -80°C. Subsequently, cells were treated with either 1 µM AZD0156 or 0.1 µM VE-822 24 hours after seeding according to previously published data of Faulhaber et al. (20). Control cells were treated with DMSO alone (vehicle control). Irradiation was performed according to a hypo-fractioned scheme with a dose of 1 x 5 Gray (Gy) 3 hours after treatment with smKI followed by a second dose of 1 x 5 Gy 24 hours later using an ISOVOLT Titan X-ray generator (GE, Ahrensburg, Germany). Cells and supernatant were collected for analysis 48 hours post-RT. The treatment scheme is displayed in **Figure 1**.

### 2.3 Cell death analysis

Cells were seeded in cell culture flasks (T25) in DMEM (10% FBS, 1% Pen/Strep). Cell number was adjusted to reach confluence of 50 – 80% during experiment. After 24 hours post-seeding, cells were treated according to our standard setting in this study (**Figure 1**). After 48 hours post-RT, supernatant and cells were harvested and stained with 0.75 μL/mL of AnnexinV-FITC (1 mg/mL; GeneArt, Regensburg, Germany), and 1.0 μL/mL of Propidium iodide (Pi) (1 mg/mL; Sigma-Aldrich, Munich, Germany) in Ringer’s solution (Fresenius, Bad Homburg, Germany) and incubated for 30 min at + 4°C. Subsequently the staining agent was removed, and the cells were centrifuged and resuspended in PBS + 2% FBS for flow cytometry measurement at Cytoflex flow cytometer (Cytoflex, Beckman Coulter, Brea, CA, USA). The Kaluza analysis software (Beckman Coulter, Brea, CA, USA) was used for gating and data analysis.

### 2.4 Immune checkpoint marker expression analysis

Cells were seeded in T25 flasks at densities of 200 000 cells or 300 000 cells. Higher densities were chosen for conditions that included irradiation. Cells and supernatant were collected for analysis 48 hours after the last treatment. Cells were either left unstained in FACS buffer without antibodies or stained with FACS buffer (PBS, Sigma-Aldrich, Munich, Germany; 2% FBS; 2mM EDTA (Carl Roth, Karlsruhe, Germany) containing the following antibodies: PD-L1-BV605 (BioLegend, Cat#329724), PD-L2-APC (BioLegend, Cat# 345508), ICOS-L-BV421(BD Bioscience, Cat#564276), OX40-L-PE (BioLegend, Cat#326308), HVEM-APC (BioLegend, Cat#318808), CD70-FITC (BioLegend, Cat#355106), CD137L-BV421 (BioLegend, Cat#311508). Staining was performed for 30 minutes at +4°C in the dark. Following staining, cells were washed once with FACS buffer and resuspended in FACS buffer for analysis using a CytoFLEX S flow cytometer (Beckman Coulter, Brea, CA, USA). Data was analyzed using Kaluza Analysis software (Beckman Coulter, Brea, CA, USA).

### 2.5 Cytokine analysis with Enzyme linked immunosorbent assay

Cytokine secretion was quantified 48 hours after the last treatment using the Multiplex ELISA Kit V-Plex Proinflammatory Panel 1 (human; K15049D) from Meso Scale Discovery (MSD) targeting the following cytokines: IFN-y, IL-1b, IL-2, IL-4, IL-6, IL-8, IL-10, IL-12p70, IL-13 and TNF-alpha. Samples were diluted (1:50) and the assay was done as per the manufacturer’s manual 18094-v5-2020Jan. The plate was read using MESO QuickPlex SQ 120 (MSD, Rockville, MD, USA) and analyzed using Discovery Workbench Software the plotted into graphs using GraphPrism. Samples were collected in four independent experiments (n = 4) and the mean values + standard deviation (SD) were used for statistical analysis.

### 2.6 RNA isolation and sequencing of HNSCC cell line Cal-33

Cells were seeded in 6-well plates at densities of 35 000 and 70 000 cells. Higher densities were chosen for conditions that included irradiation. Supernatant was discarded 48 hours after the last irradiation and HNSCC cells were harvested using 1 mL Trizol (TRIzol Reagenz, Invitrogen, Waltham, Massachusetts, USA) per well followed by RNA isolation via chloroform-phenol extraction. Subsequently, RNA purification as well as DNAse digestion was performed using a RNeasy mini Kit from Qiagen.

Sequencing libraries were prepared with SMARTer stranded RNA (poly A selection) and sequenced on an Illumina NovaSeq 6000 (2 x 150 bp) by Macrogen. Raw reads were pseudo-aligned to the human reference transcriptome (Ensembl; Homo sapiens version 100) using Kallisto (version 0.46.0), and MultiQC (v.18; including the tools of FastQC, Samtools, Picard, GATK, RSeQC, SnpEff and DRAGEN) were be used to check the quality of the alignment. All subsequent analyses were conducted using the statistical computing environment R (version 4.1.0), RStudio (version 1.4.1717) and Bioconductor (version 3.13). Differential gene expression was assessed with edgeR after removal of low-expressed genes. Genes with a log2foldchange > 1 and false discovery rate of ≤ 0.05 were determined as differentially expressed.

## 3 Results

### 3.1 RT combined with ATRi shows the highest toxicity in HNSCC cells, regardless of the HPV status

**Figure 2:**
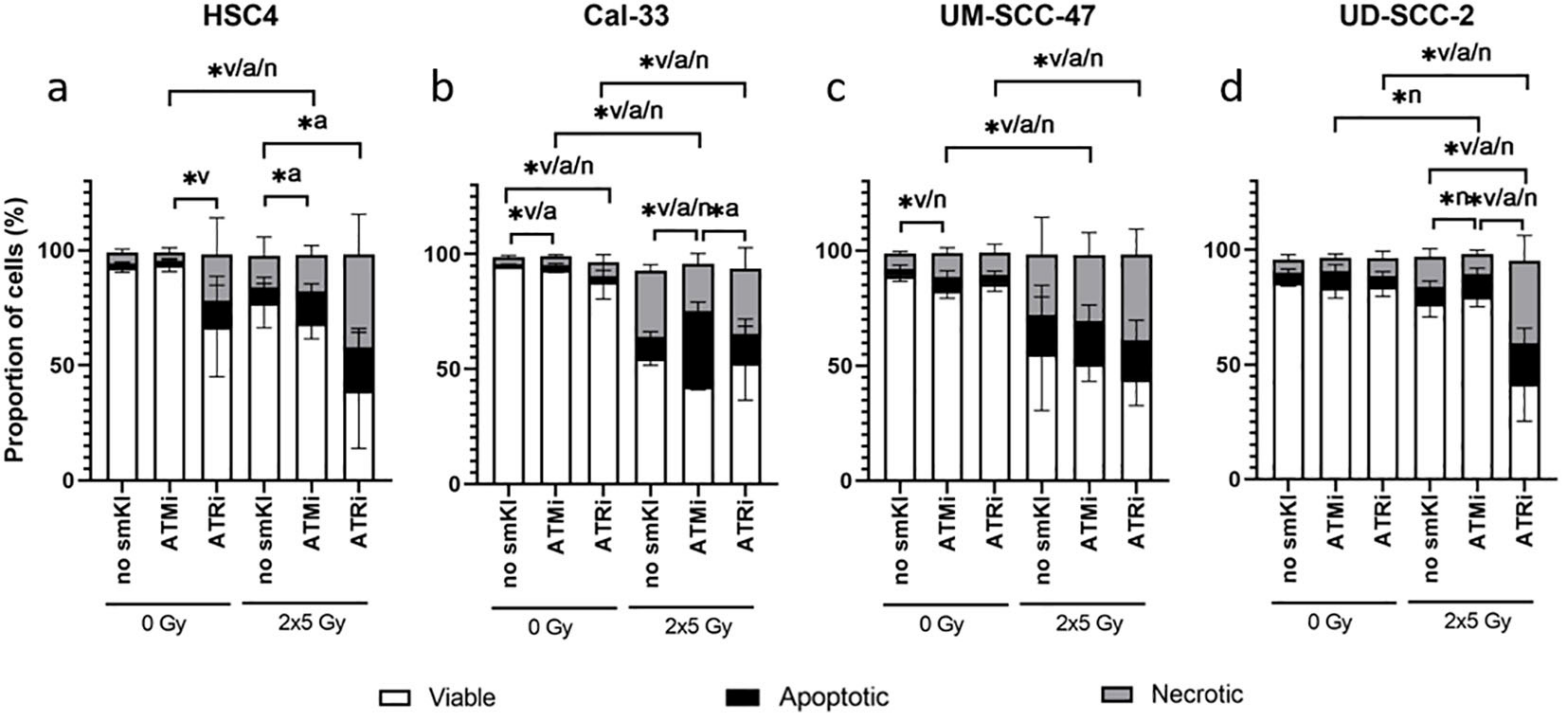
Cell death analysis of HNSCC treated with ATMi or ATRi w/o 2x5Gy RT. RT combined with ATRi shows the highest toxicity in HNSCC cells, regardless of the HPV status. 48 hours post-RT, HPV-negative HSC4 **(A)** and Cal-33 **(B)** cells as well as HPV-positive UM-SCC-47 **(C)** and UD-SCC-2 **(D)** cells were identified as viable (AnnexinV-, PI-), apoptotic (AnnexinV+, PI-) or necrotic (AnnexinV+, PI+). Percentages of viable, apoptotic, and necrotic cells are shown as stacked bars representing the mean ± SD. A one-tailed Mann-Whitney U test was performed to compare the different treatment approaches within one cell line: *p ≤ 0.05, n=4.

We started to investigate the toxicity of the examined smKI AZD0156 (ATMi) and VE-822 (ATRi) by treating all four cell lines with either smKI or RT alone, as well as their combination. Subsequently, we analyzed cell survival and cell death forms by quantifying the percentages of viable, apoptotic and necrotic HNSCC cells following the different treatment approaches (**Figure 2**).

In two of the four cell lines, treatment with smKI alone showed no enhanced toxicity (**Figures 2A, D**). However, for Cal-33, inhibition of ATM or ATR alone resulted in a slight, but significantly increased percentage of apoptotic or necrotic cells, respectively (**Figure 2B**). In three of the four cell lines, hypo-fractionated RT (2x5Gy) primarily induced cell death, and its effects were tendentially further augmented with combined treatment (**Figures 2B–D**). For HSC4 and UD-SCC-2 cells, the combined treatment with RT and ATRi resulted in the highest number of dead cells with a higher prevalence of necrotic as opposed to apoptotic cells (**Figures 2A, D**). Taken together, the impact of the treatment approaches showed cell line-specificity, regardless of HPV status. Notably, the combined treatment of RT and ATRi showed the highest induction of cell death, with the majority of cells undergoing necrotic cell death.

### 3.2 Upregulation of immunostimulatory ICOS-L and CD137-L on HNSCC cells by combining RT with ATRi

**Figure 3:**
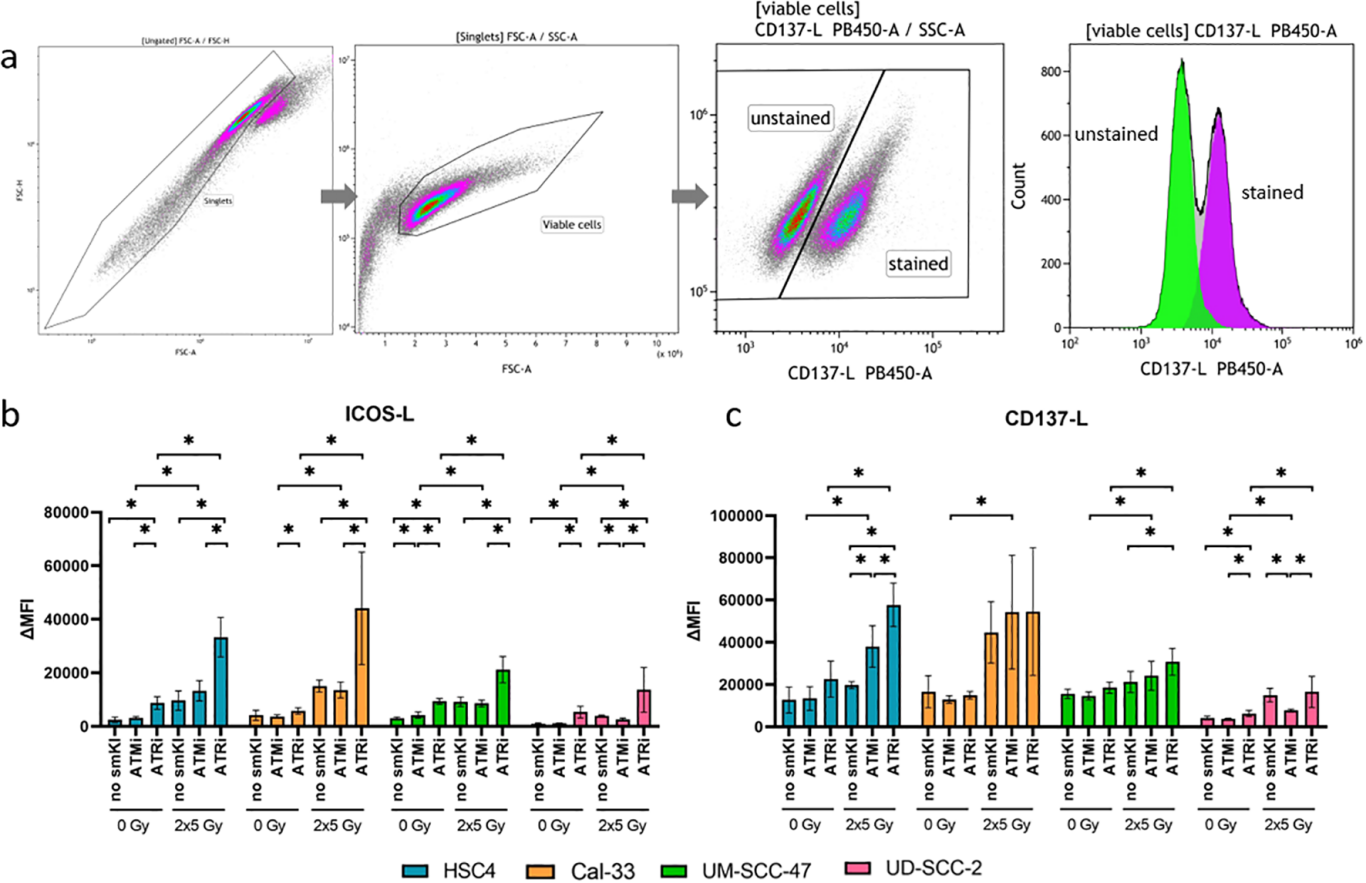
Flow cytometric analysis of immune checkpoint surface marker on HNSCC after ATMi or ATRi w/o 2x5Gy RT. Combining RT with ATRi leads to the upregulation of immunostimulatory ICOS-L and CD137-L on HNSCC cells, irrespective of the HPV status. 48 hours after the last treatment, the HNSCC cells were harvested and the expression of immunostimulatory ICMs ICOS-L and CD137-L was examined by flow cytometry. The gating strategy is presented in **(A)** ICM expression is presented as ΔMFI (delta of mean fluorescence intensity of stained samples – background fluorescence) **(B, C)**. The combined treatment of RT and ATRi increased ICOS-L expression in all four cell lines compared to ATRi or RT alone, as well as in comparison to RT combined with ATMi **(B)**. Dual treatment with RT and ATMi tended to even decrease ICOS-L surface expression compared to RT or RT with ATRi **(B)**. RT and ATRi led to enhanced expression of immunostimulatory CD137-L in HPV-negative HSC4 cells compared to ATRi or RT alone, or the combined treatment of RT and ATMi **(C)**. In HPV-positive UM-SCC-47 and UD-SCC-2 cells, the combination of RT and ATRi also demonstrated higher surface expression of CD137-L compared to RT alone or treatment with RT and ATMi, respectively **(C)**. A one-tailed Mann-Whitney U test was performed to compare the different treatment approaches within one cell line: *p ≤ 0.05, n=4.

Anti-tumor response is triggered or suppressed by the expression of immunostimulatory or -suppressive surface checkpoint molecules (ICMs) on tumor cells. We recently showed that combining RT with chemotherapy leads to increased expression of immunostimulatory ICMs ICOS-L and CD137-L on HNSCC cells (26). Subsequently, we now aimed to assess the immunomodulatory effects of combing RT with smKI via the expression of immunostimulatory and -suppressive ICMs on HNSCC cells. Therefore, we treated all four cell lines with either smKI or RT alone or their combination and examined the expression of ICMs on the cell surface of the HNSCC cells 48 hours post-RT (**Figure 3**). The gating strategy is exemplarily shown for CD137-L in **Figure 3A**. The combined treatment of RT and ATRi increased ICOS-L expression in all four cell lines compared to ATRi or RT alone, as well as in comparison to RT combined with ATMi. Conversely, dual treatment with RT and ATMi tended to even decrease ICOS-L surface expression compared to RT or RT with ATRi (**Figure 3B**). Additionally, RT and ATRi led to enhanced expression of immunostimulatory CD137-L in HPV-negative HSC4 cells compared to ATRi or RT alone, or the combined treatment of RT and ATMi. In HPV-positive UM-SCC-47 and UD-SCC-2 cells, the combination of RT and ATRi also demonstrated higher surface expression of CD137-L compared to RT alone or treatment with RT and ATMi, respectively (**Figure 3C**).

**Figure 4:**
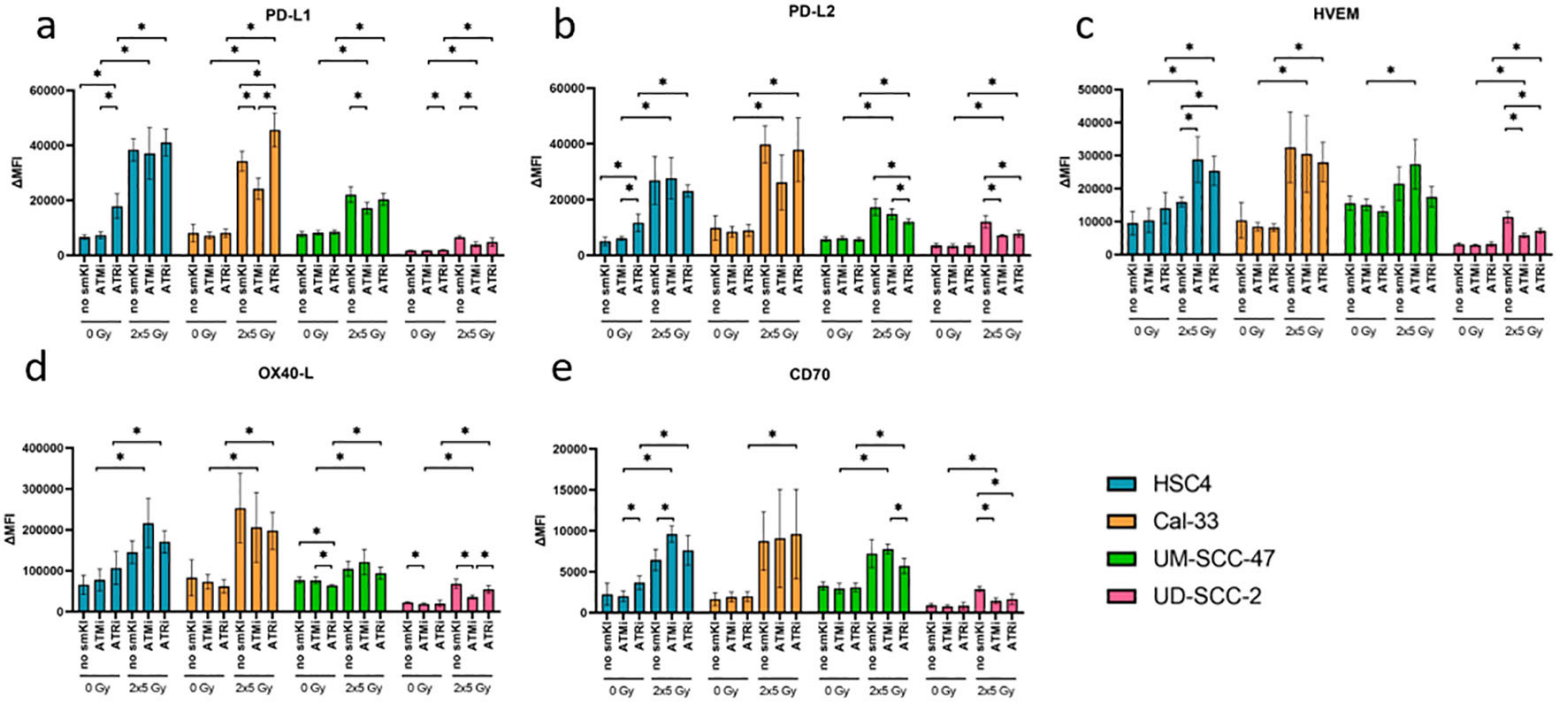
Flow cytometric analysis of immune checkpoint surface marker on HNSCC after ATMi or ATRi w/o 2x5Gy RT. Combining RT with ATRi leads to decreased expression of PD-L2 in HPV-positive cell lines UM-SCC-47 and UD-SCC-2. 48 hours after the last treatment, the HNSCC cells were harvested and the expression of the immunosuppressive ICMs PD-L1 **(A)**, PD-L2 **(B)**, HVEM **(C)**, OX40-L **(D)** and CD70 **(E)** was examined by flow cytometry. ICM expression is presented as ΔMFI (delta of mean fluorescence intensity of stained samples – background fluorescence) **(A–E)**. Combined treatment with RT and ATRi had no significant impact on the expression of PD-L1 compared to RT alone, except for the Cal-33 cell line, where it resulted in upregulation. However, it led to decreased expression of PD-L2 **(B)** in both HPV-positive cell lines and decreased expression of HVEM **(C)** in one HPV-positive cell line. The impact of the treatment approaches on the expression of OX40-L **(D)** and CD70 **(E)** showed cell line-specificity, regardless of the HPV status. A one-tailed Mann-Whitney U test was performed to compare the different treatment approaches within one cell line: *p ≤ 0.05, n=4.

We further checked the expression of immunosuppressive ICMs PD-L1, PD-L2, HVEM, and immunostimulatory ICMs OX40-L and CD70. Here, combined treatment with RT and ATRi had no significant impact on the expression of PD-L1 compared to RT alone, except for the Cal-33 cell line, where it resulted in upregulation. Dual treatment with RT and ATMi, however, resulted in decreased PD-L1 expression in three out of four cell lines in comparison to RT alone (**Figure 4A**). Interestingly, we found decreased expression of PD-L2 in both HPV-positive cell lines and decreased levels of HVEM expression in one HPV-positive cell line following treatment with RT and ATRi in comparison to RT (**Figures 4B, C**). The impact of the treatment approaches on the expression of OX40-L and CD70 showed cell line-specific patterns, regardless of HPV status (**Figures 4D, E**). Taken together, combining RT and ATRi resulted in the upregulation of immunostimulatory surface molecules ICOS-L and CD137-L while simultaneously leading to the downregulation of the immunosuppressive PD-L2.

### 3.3 Enhanced secretion of proinflammatory cytokines IL-8, IL-6, TNF-α, IL-13, IL-12, IL-1β and IL-10 by combining RT with ATRi

**Figure 5:**
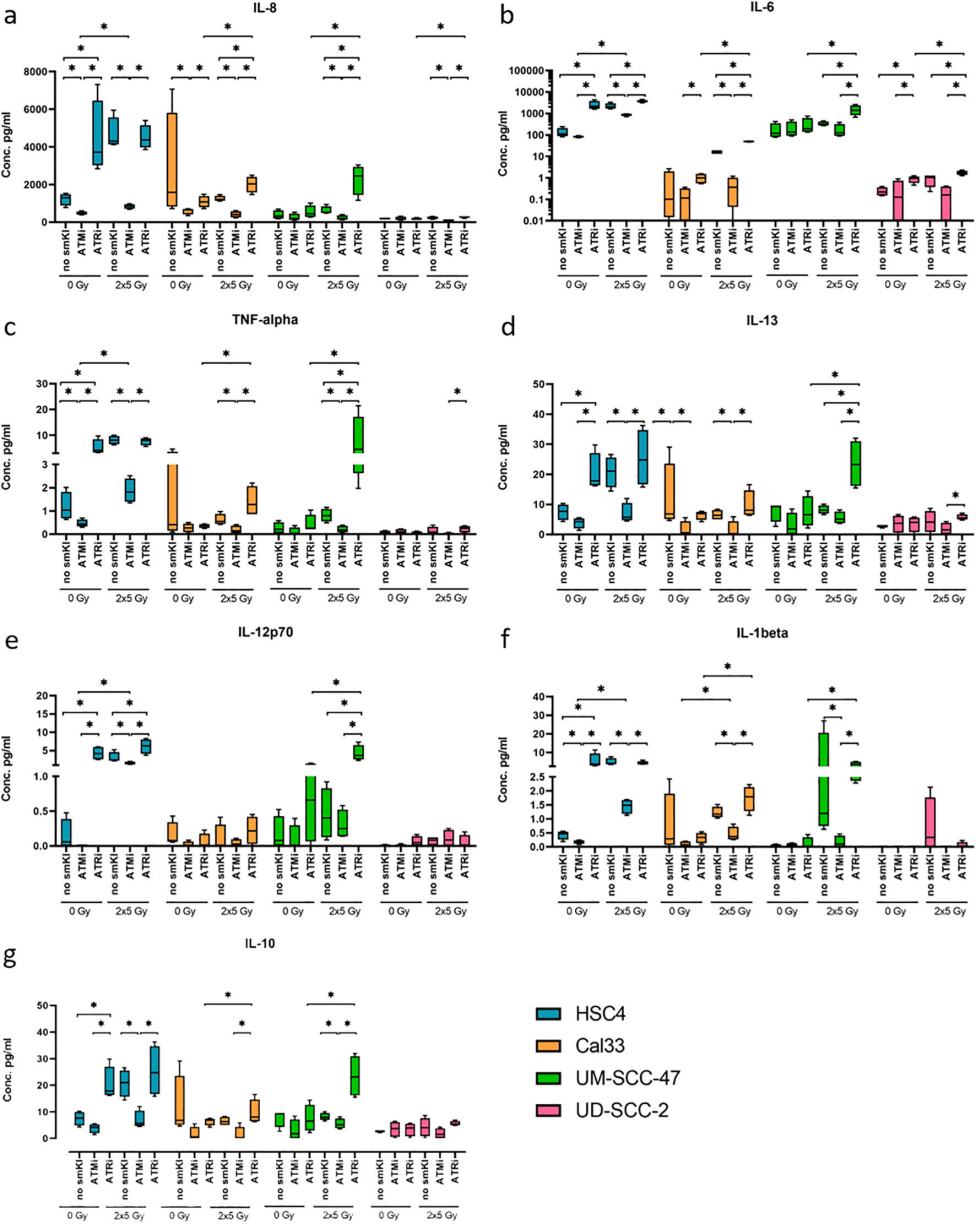
Multiplex ELISA analysis (MSD) of seven cytokines released by HNSCC after ATMi or ATRi w/o 2x5Gy RT. Combining RT with ATRi enhances the secretion of proinflammatory cytokines IL-8, IL-6, TNF-α, IL-13, IL-12, IL-1β, IL-10 by HNSCC cells, independent of their HPV status. Cytokine secretion was quantified 48 hours after the last treatment. Samples were collected in four independent experiments (n = 4) and the mean values + standard deviation (SD) were used for statistical analysis. The combined treatment of RT and ATRi led to an increased secretion of immunostimulatory cytokines IL-8 **(A)**, IL-6 **(B)**, TNF-α **(C)**, IL-13 **(D)** compared to the combination of RT and ATMi in all four cell lines, along with increased levels of IL-12p70 **(E)** and IL-1β **(F)** in two or three out of four cell lines, respectively. Regarding immunoinhibitory cytokines, combined treatment with RT and ATRi led to increased levels of IL-10 in three of the four cell lines **(G)**. A one-tailed Mann-Whitney U test was performed to compare the different treatment approaches within one cell line: *p ≤ 0.05, n=4.

Having observed that dual treatment with RT and smKI alters the expression of immune-related surface markers on the HNSCC cells, we next examined whether cytokine secretion is influenced by the combination of RT and smKI. Therefore, we treated the two HPV-negative and two HPV-positive HNSCC cell lines with either RT or smKI alone or their combination and measured cytokine levels 48 hours post-RT. We here assessed a panel of pro- and anti-inflammatory cytokines known to play a role in either pro- or anti-tumoral responses, including IL-8, IL-6, TNF-α, IL-13, IL-12p70, IL-10, IL-4, IL-2, IL-1β and IFN-γ (**Figure 5**). We did not detect any significant differences in the secretion of IL-4, IL-2, IFN-γ in between the different treatment approaches (data not shown).

Notably, the combined treatment of RT and ATRi led to an increased secretion of the cytokines IL-8, IL-6, TNF-α, and IL-13 compared to the combination of RT and ATMi in all four cell lines, along with increased levels of IL-12p70 and IL-1β in two or three out of four cell lines, respectively. Dual treatment with RT and ATMi, however, demonstrated a decrease in the levels of respective cytokines (**Figures 5A–F**). Additionally, combining RT and ATRi increased the secretion of proinflammatory cytokines IL-8, IL-6 and IL-12p70 compared to RT alone (**Figures 5A, B, E**). Regarding key anti-inflammatory cytokines, we checked for the secretion of IL-10, IL-4, and IL-2. Here, combined treatment with RT and ATMi led to decreased levels of IL-10 in three of the four cell lines whereas RT + ATRi increased IL-10 levels (**Figure 5G**). Overall, these data indicate that dual treatment of RT and ATRi induces an upregulation of cytokines IL-8, IL-6, TNF-α, IL-13, IL-12p70, IL-1β and IL-10, independent of the HPV status, whereas combining RT and ATMi is more likely to reduce the expression of respective cytokines.

Particularly HPV-independent Cal-33 cells showed mainly RT-induced toxicity and limited influence by the combination of ATMi or ATRi. The analysis of the expression of immune-modulating surface markers on the HNSCC also shows for Cal-33 that on the one hand RT mainly contributes to the regulation of these markers, but on the other hand the expression level of PD-L1 and PD-L2 on the cell surface contributes to a significant reduction of these markers by the additional administration of ATMi to RT. We decided to carry out an RNAseq analysis for Cal-33 to mainly elucidate whether differences between RT plus ATMi versus RT plus ATRi occur on the transcriptome level.

### 3.4 Strong down-regulation of immune checkpoint molecules and cytokines on transcript level after RT + ATMi treatment of Cal-33

**Figure 6:**
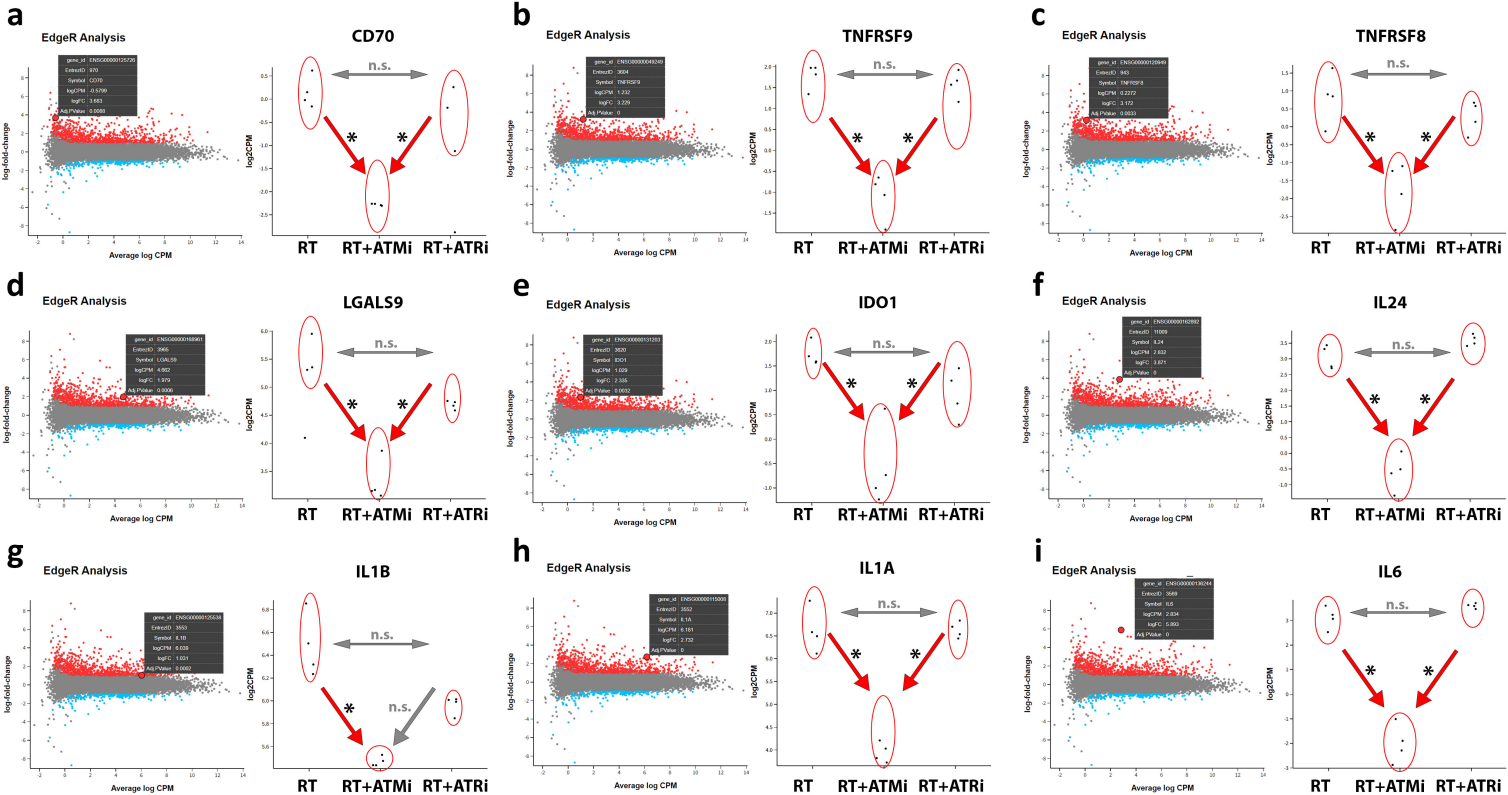
RNAseq analysis of treated Cal-33. Modulation of specific transcripts based on a RNAseq analysis of treated HPV-negative Cal-33 cells (RT vs. RT+ATMi vs. RT+ATRi). Cells were treated according to the standard scheme in this study (see **Figure 1**). Immune-modulating cell surface marker such as CD70 **(A)**, TNFRSF9 known as CD137-L **(B)**, TNFRSF8 known as CD30 **(C)** and LGALS9 known as Galectin-9 **(D)** were significantly downregulated by the combination of RT and ATMi AZD0156. Further, soluble factors such as IDO1 **(E)** and immune-related cytokines such as IL-24 **(F)**, IL-1b **(G)**, IL-1a **(H)** and IL-6 **(I)** were also downregulated by the combination of RT + ATMi. Data representing 4 independent replicates. All transcripts were declared as significant when log-(foldchange) > ± 1 was determined and the adjusted p-value was ≤ 0.050. All transcripts were declared as significant (*) when log-(foldchange) > ±1 was determined and the adjusted p-value was ≤0.050.

It was hypothesized that concomitant treatment of hypo-fractionated RT and either ATM inhibitor or ATR inhibitor will lead to radiosensitization and immune-modulation preferably in HPV-negative cancer cells. However, at the protein level, we did not observe the significant influence of smKI that we had anticipated in HPV-negative Cal-33 cells. Therefore, we performed RNAseq analyses to reveal possible differences on the mRNA level. Initially, we examined the mRNA expression of previously tested immune checkpoint markers, as well as modulators of the immune system such as interleukins (**Figure 6**). To understand the mechanisms that might contribute to the effectiveness of ATRi or ATMi as radiosensitizers, we focused on comparing cells treated with radiation therapy (RT) alone to those treated with a combination of RT and either ATMi or ATRi.

The mRNA analysis revealed that combinatory treatment of RT and ATM inhibitor leads to a significant down-regulation of several mRNAs of central immune modulating proteins. CD70 and CD137-L were significantly down-regulated after RT + ATMi treatment of Cal-33 compared to RT and RT + ATRi, but no significant difference was detected between RT and RT + ATRi. This pattern was also observed for immune modulators CD30 and Galectin-9. Besides these surface markers, our RNAseq analysis showed also a down-regulation of Indoleamine 2,3-Dioxygenase 1 (IDO-1) and interleukin 24, IL-1b, IL-1a and IL-6. The combination of ATM inhibitor with RT consistently led to down-regulation compared to RT alone and the combination of RT with ATR inhibition, except for IL-1b. For IL-1b, no difference was observed between the RT + ATMi and RT + ATRi treatments.

**Figure 7:**
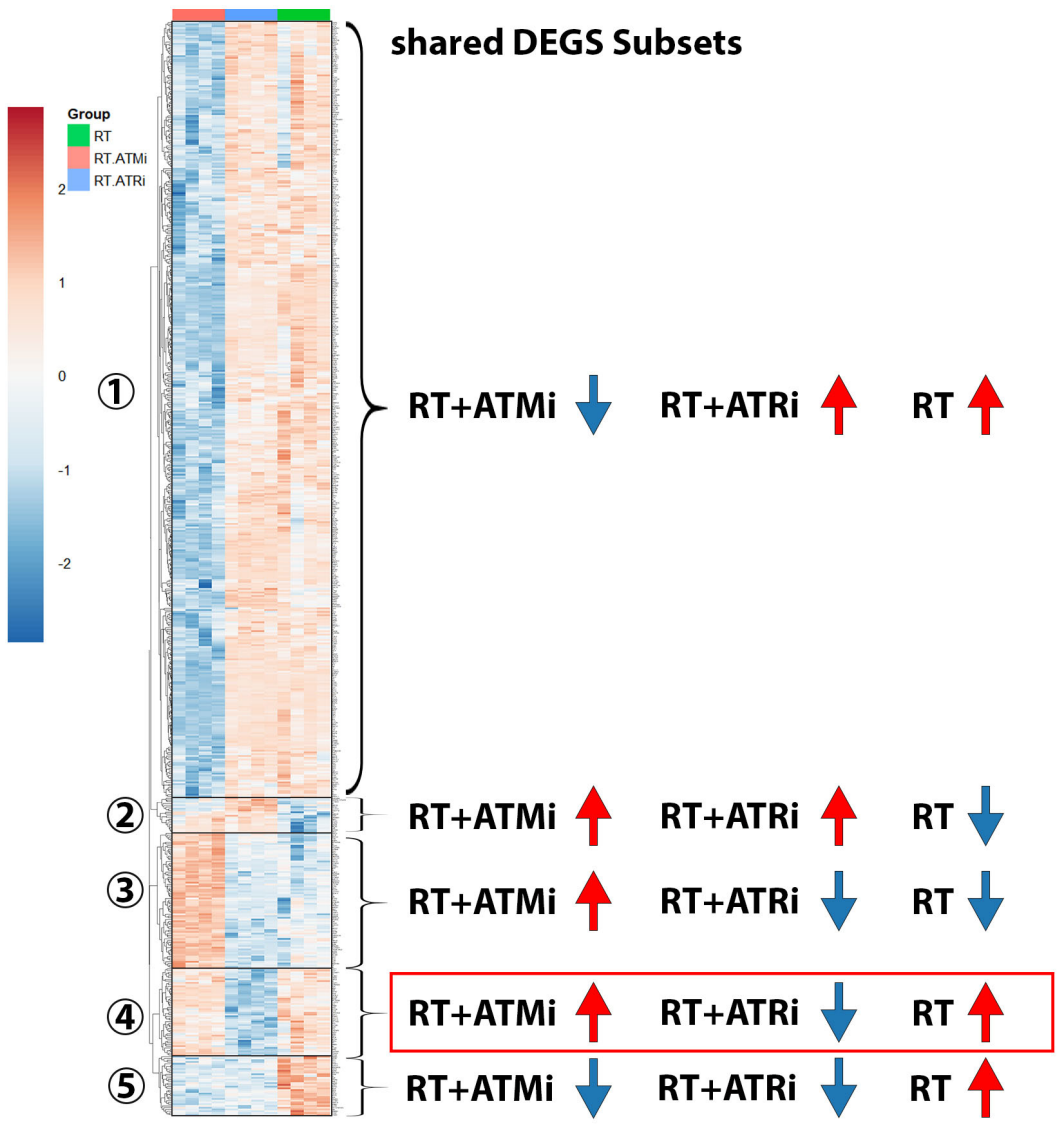
Heatmap including subsets of shared DEGs of Cal-33 treated with RT, RT+ATMi or RT+ATRi for 48h. First subset shows all genes down-regulated only in RT+ATMi and includes 690 genes. Second subset shows all genes down-regulated only by RT (20 genes). Third subset shows all down-regulated genes by RT and RT+ATRi (188 genes). Forth subset shows all genes down-regulated only in RT+ATRi treated Cal-33 (50 genes). Fifth subset shows all genes down-regulated by both combinations RT+ATMi and RT+ATRi (34 genes). Data representing 4 independent replicates. All transcripts were declared as significant when log-(foldchange) > ± 1 was determined and the adjusted p-value was ≤ 0.050. Representative MA plots show comparison of RT vs. RT+ATMi treated Cal-33 mRNA expression.

These immune-specific mRNAs demonstrate a strong immune-inhibitory influence of ATM inhibitor AZD0156 on the transcriptome. In general, the analysis of all shared differential expressed genes (DEGs) showed that there is a big subset of down-regulated genes in RT + ATMi treated Cal-33 (**Figure 7**).

In total 982 shared DEGs were detected in Cal-33 after treatment with RT, RT + ATMi and RT + ATRi (**Figure 7**). The biggest group (**Figure 7**-subset 1) of DEGs was down-regulated only by the treatment of RT + ATMi in Cal-33. Furthermore, we identified subsets of genes that were specifically down-regulated only after RT (**Figure 7**-subset 2) or down-regulated by RT + ATRi (**Figure 7**-subset 4) alone. There was also a group of genes down-regulated by the combination of RT + ATMi and RT + ATRi (**Figure 7**-subset 5) and, in contrast to the first subset, we also could identify 188 genes shared in all conditions but solely up-regulated by RT + ATMi (**Figure 7**-subset 3).

**Figure 8:**
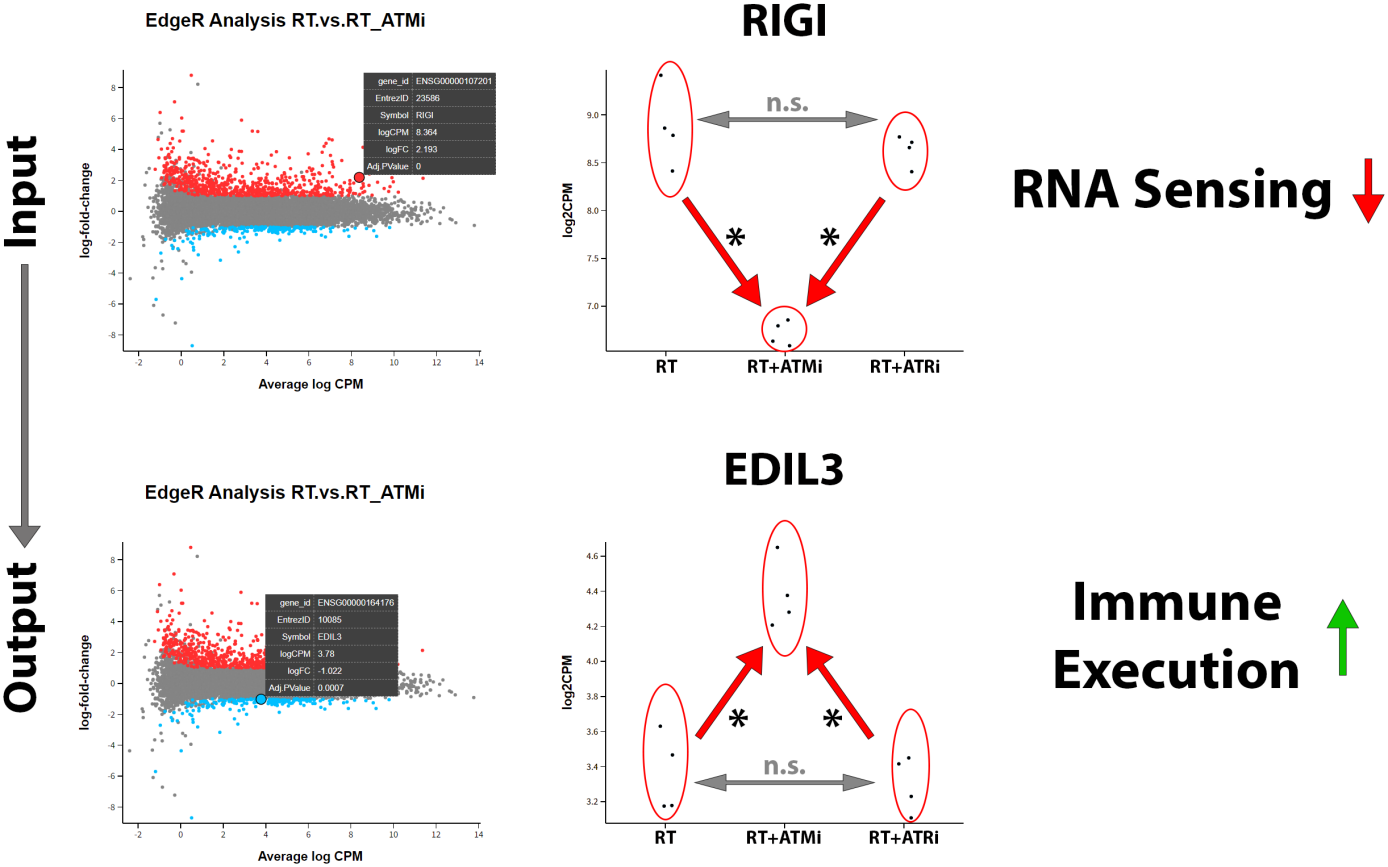
Treatment of Cal-33 with a combination of hypo-fractionated RT and ATM inhibitor AZD0156 leads to significant downregulation of nucleic acid sensor RIG-1 and significant up-regulation of immune-execution protein EDIL3. RIG1 plays a central role in recognition and processing of dsRNA (double-stranded RNA) in mammalian cells and is strongly down-regulated by the combination of ATM inhibitor AZD0156 with RT compared to RT or RT+ATRi treatment alone. In contrast to this, EDIL3, which is associated with immune exclusion and poor prognosis, is highly up-regulated by this combination, without differing between RT or RT+ATRi treatment in Cal-33. All transcripts were declared as significant (*) when log-(foldchange) > ±1 was determined and the adjusted p-value was ≤0.050.

In our analysis of mRNA expression influenced by RT or the combination with smKI targeting the DDR we were able to detect another interesting target. Our analysis revealed that, in addition to the previously mentioned immune checkpoint markers and modulating cytokines, the combination of RT + ATMi also leads to a significant down-regulation of the antiviral innate immune response receptor retinoic acid-inducible gene I (RIG-I), a central nucleic acid sensor playing a role in nucleic acid sensing pathways (27). According to this finding, we identified EGF-like repeat and discoidin I-like domain-containing protein 3 (EDIL3), a protein which is associated with immune exclusion leading to nonresponse to immune checkpoint blockage and therefore correlated with poor prognosis in several malignancies (28). EDIL3 was significantly up-regulated via combination of ATM inhibitor AZD0156 and hypo-fractionated RT (**Figure 8**).

The mRNA analysis of Cal-33 demonstrated that immune-related surface markers were significantly down-regulated after treatment with both RT + ATMi but not after RT alone or RT + ATRi, respectively. Furthermore, we identified a decrease in immune-modulating cytokines and, interestingly, the down-regulation of a central protein of the nucleic acid sensing pathway RIG-I. Additionally, we detected the up-regulation of EDIL3 in Cal-33 treated with RT + ATMi. So far, there is just limited knowledge of the function of EDIL3 in HNSCC.

## 4 Discussion

HNSCC has been shown to compromise immunosuppressive characteristics such as low absolute lymphocyte numbers, diminished NK cell activation and low antigen-presenting function (28), highlighting the requirement of enhanced immune-related anti-tumor response.

We hypothesized that combining RT with smKI not only amplifies the effects of irradiation, previously shown by Faulhaber and colleagues (21), but also alters the immunogenetic characteristics of HNSCC in dependence of the HPV status. Additionally, we expected that inhibiting two distinct key kinases involved in DDR, namely ATM or ATR, would result in differing impacts on the immunogenicity of the HNSCC cells. This might also hold as an explanation for the frequent use of ATR inhibitors in currant clinical trials in contrast to ATM inhibitors. While numerous studies have already examined the effectiveness of ATM and ATR inhibition alone or in combination with RT for different cancer types, there remains a notable gap in directly comparing their impact on the immunogenicity of HNSCC cells (16–19). Additionally, existing literature primarily focuses on single doses typical of daily RT, such as 2 Gy, or single high doses like 10 Gy. Building upon prior research, we employed a hypo-fractionated RT regimen of 2x5 Gy, known for its clinically relevant immune-modulating properties after RT (29) and despite improvements in HPV-positive HNSCC outcomes, HPV-negative HNSCC outcomes have stagnated for decades, leading to interest in hypofractionation as a potential treatment, with ongoing clinical trials exploring its feasibility and safety. Consequently, we directly compared the influence of combined treatment involving ATMi + hypo-fractionated RT or ATRi + hypo-fractionated RT on both protein and mRNA levels.

### 4.1 The cytotoxic effect of RT is improved by combining RT with ATMi or ATRi

We first showed that single treatment of HNSCC cells with smKI alone resulted in either no or only slight toxicity in all cell lines. Interestingly, even if not yet significant, treatment with ATRi induced necrosis to a great extent in HSC4 then in Cal-33. A mutation in the PTEN gene of HSC4 and findings of a strong expression of TIPE3, an apoptosis-regulating protein, could be the reasons for an intensified induction of necrosis (30, 31). Overall, hypo-fractioned RT (2x5 Gy) had the most significant impact on inducing apoptotic or necrotic cell death in HNSCC cells, regardless of their HPV status. Our results indicate that both radiosensitivity and sensitivity to combined treatments of HNSCC cells regarding cell death induction are rather associated with specific cell line characteristics than correlated with HPV status.

In three out of four cell lines, the effects of RT were tendentially further augmented by combining RT with ATMi or ATRi. Specifically, ATMi + RT primarily led to apoptotic cell death. Interestingly, inhibition of ATR + RT tendentially resulted in a higher percentage of necrotic cell death compared to ATMi + RT. Apoptosis, also known as programmed cell death, is part of physiological tissue homeostasis within an organism. Apoptotic cells are typically eliminated through an anti-inflammatory pathway (32, 33). On the other hand, necrotic cells are characterized by the loss of cell membrane integrity, resulting in the secretion of DAMPs that trigger inflammation and immune response. Thus, the immunogenicity of cancer cells undergoing necrotic cell death is augmented (34). An immunogenic form of cell death is the favorable event in cancer treatment as it induces anti-tumor immune response in support of treatment efficacy (35).

In three out of four cell lines, combined treatment of RT + ATRi not only resulted in the highest induction of cell death but also the highest percentage of cells undergoing necrosis compared to RT alone or RT + ATMi. These findings align with previous studies demonstrating the radiosensitizing effects of ATR inhibitor VE-822 *in vitro* for HNSCC (21, 36). We assume that these cell line-specific differences are based in the different potential of all cell lines for adequate DNA repair. There is new data published showing that HSC4, Cal-33, UM-SCC-47 and UD-SCC-2 express different amounts of RAD51, a central protein of the HR system. This might also explain the ambivalent effects of CHK1/2 inhibitors on both, HPV-positive as well as HPV-negative, cell lines (4). This indicates that augmentation of RT with ATRi might be beneficial to enhance the immunogenicity in HNSCC treatment, potentially stimulating immune-related anti-tumor response. However, cell death is a multifactorial process that can be further divided into several subtypes that differ from each other in terms of stimuli, pathways and biological properties such as immunogenicity (32, 35, 37, 38). Hence, future vaccination assays in immunocompetent mice are required to validate the immunogenicity of cell death forms following combined treatments with RT + ATMi and RT + ATRi (39).

### 4.2 Addition of ATRi to RT reduces the expression of immunosuppressive checkpoint molecules triggered by RT

Dying cells, as well as cells treated without undergoing cell death, can significantly impact the immune system by altering their characteristics (35, 40). This may lead to changes in the expression of cell-surface proteins, or the secretion of cytokines related to immunity. Furthermore, HNSCC has been shown to have a rather high tumor mutational burden (TMB) that is associated with a better response to immune checkpoint inhibitors (41). Hence, we next examined the impact of combined treatment on the expression of both immunosuppressive (e.g. PD-L1, PD-L2) and immunostimulatory (e.g. ICOS-L, CD137-L) cell surface proteins in HNSCC cells.

In alignment with our observations regarding cell death induction, the impact of the combined treatment on the expression and secretion of immune-related proteins is more influenced by the specific characteristics of the cell line rather than being correlated with HPV status.

To date, the most prominent approach in immunotherapy of HNSCC focuses on targeting the PD-1/PD-L1 axis. PD-1 is expressed on T cells where its interaction with its ligands PD-L1 and PD-L2 initiates T cell inhibition, contributing to cancer immune evasion. The introduction of PD-1 blockades through Pembrolizumab and Nivolumab has established new treatment standards for recurrent or metastatic HNSCC (42–44).

Therefore, we investigated the expression of immunosuppressive markers PD-L1 and PD-L2, along with HVEM, following treatment in HNSCC cells. Notably, hypo-fractioned RT led to an upregulation of all these markers. For HVEM, combining RT with smKI showed cell line-specific effects that precluded definitive conclusions regarding potential new treatment strategies. Combining RT with ATMi tendentially decreased the expression of PD-L1 and PD-L2 compared to RT treatment alone, though these effects did not reach statistical significance.

Interestingly, RT + ATRi significantly reduced PD-L2 expression in HPV-positive HNSCC cell lines compared to single RT treatment, while having no significant impact on PD-L2 expression in HPV-negative HNSCC cells. Additionally, RT + ATRi did not significantly alter PD-L1 expression compared to RT treatment alone, except for the Cal-33 cell line, where it resulted in upregulation. These findings indicate that the addition of ATRi to RT tends to reduce the expression of ICMs triggered by RT, potentially contributing to immune-related tumor response. However, the possibility of upregulating immunosuppressive PD-L1 in the cells should be considered, including prevention by established PD-1 blockers such as Pembrolizumab or Nivolumab.

We next examined the expression of immunostimulatory ICMs on the HNSCC following combined treatment. For CD70 and OX40-L, hypo-fractioned RT mainly led to an upregulation of the expression. Interestingly, RT induced a greater upregulation of both ICMs in HPV-negative HNSCC cell lines compared to HPV-positive cell lines. Since treating HPV-negative HNSCC remains a challenge, the heightened immune response observed here may contribute to improved treatment outcomes. However, the effect of adding smKI to RT on the expression of CD70 and OX40-L exhibited strong cell line-specificity, making it difficult to predict an overall anti-tumor immune response. Consequently, we turned our attention to two additional immunostimulatory ICMs we examined: CD137-L and ICOS-L.

The growing interest in endogenous tumor-infiltrating lymphocytes (TILs) as prognostic biomarkers in different cancer types, such as HNSCC, reveals new treatment approaches (45, 46). Naturally occurring or generated T cells targeting cancer antigens showed success in controlling disease progression in HPV-associated cancers (47) (47, 48). While the blockade of the inhibitory checkpoint PD-1/PD-L1 has become a well-established treatment strategy for several cancer entities, including recurrent/metastatic HNSCC, relevant relapse rates post-treatment require the exploration of additional immune checkpoints and cytokines involved in stimulating TILs (43, 49, 50). One promising candidate here is CD137-L, whose expression on immune cells not only initiates T cell proliferation, activation and survival but also induces NK cell activation (51, 52). To date, the findings about using CD137/CD137-L interaction in immunotherapy are inconsistent: Some studies have not detected any additional benefits from combining the CD137 agonist Urelumab with monotherapies using cetuximab or nivolumab (53). However, Srivastava et al. have demonstrated that Urelumab enhances the immune response activated by cetuximab in HNSCC (54). Additionally, Lucido et al. showed that tumor cell expression of CD137-L enhances tumor clearance in HPV-positive HNSCC (55). Additionally, we previously demonstrated that not only CD137-L is upregulated after RT but also the release of HMGB1, a marker for immunogenic cell death (26). In our experiments, combined treatment of RT + ATRi resulted in increased expression of CD137-L compared to single RT treatment in two of four cell lines. Further, this combination treatment tendentially induced the highest levels of CD137-L expression across all four cell lines, revealing its potential to enhance immune-related anti-tumor response.

Besides CD137, preclinical and early-clinical studies revealed effectiveness of the stimulation of the inducible T cell co-stimulator (ICOS). These effects were even shown to lead to a positive synergism following anti-PD-1 treatment (50, 56, 57). ICOS belongs to the CD28 family of co-stimulatory immunoreceptors and is mainly present on the T cell surface where its interaction with ICOS-L induces T cell activation and differentiation (25, 58). Therefore, signaling through ICOS is essential for the initiation of an adaptive immune response via T cell dependent B cell response. However, Montes-Casado et al. recently showed that ICOS deficiency diminishes NK cell homeostasis, function and development, indicating its additional role in the induction of innate immunity (59). The expression of ICOS may therefore be correlated with clinical outcomes and prognosis, highlighting the importance of ICOS-L upregulation on tumor cells as a possible treatment approach. In our experiments, combined treatment of RT + ATMi tendentially decreased ICOS-L expression on the HNSCC cells compared to single RT. In contrast, RT + ATRi significantly increased ICOS-L expression in all four cell lines compared to RT alone, indicating to enhance immune-related anti-tumor response regarding both innate and adaptive immunity.

Taken together, the combined treatment of ATRi + RT led to increased levels of both CD137-L and ICOS-L on HNSCC cells, suggesting its potential to induce T cell activation. These findings suggest that triple treatment approaches incorporating RT, anti-PD-1 therapy and ATRi might further enhance anti-tumor response, particularly in patients exhibiting limited responsiveness to single anti-PD-1 approaches. These observations align with early-phase clinical trial data proposing that combining various immune checkpoint inhibitors with RT could amplify the effectiveness of HNSCC treatment (60). It is important to note that our findings are confined to *in vitro* experiments that need to be validated *in vivo*. However, Liu et al. recently showed that combining ATRi with RT not only enhanced T cell infiltration but also improved the efficacy of anti-PD-L1 therapy in colorectal cancer in mouse models with different microsatellite models (19).

### 4.3 Cytokine-release profile of RT + ATRi shows pro- and anti-inflammatory features

As previously noted, the altered immunogenicity of treated HNSCC cells may not only be indicated by changes in ICM expression but also through cytokine secretion. HNSCC is known to circumvent the growth-inhibiting effects of anti-tumor cytokines through a network of regulatory factors. For instance, HNSCC not only secretes cytokines to impact on the tumor microenvironment (TME) but also recruits immune-suppressive cells that, in turn, secrete additional pro-tumoral cytokines (61). This immunosuppressive TME is known to contribute to ineffectiveness of immunotherapy (62, 63). The constellation of the cytokinome in the TME might thereby be useful as biomarkers to predict prognoses and influence therapeutic choices (64). Therefore, we investigated the secretion of pro- and anti-inflammatory cytokines known to influence either pro- or anti-tumoral responses, including IL-8, IL-6, TNF-α, IL-13, IL-12p70, IL-10, IL-4, IL-2, IL-1β, and IFN-γ.

Consistent with our findings on cell death induction and surface expression of ICMs, the combination of RT + ATMi exhibited predominantly anti-inflammatory patterns, whereas RT + ATRi resulted in increased pro-inflammatory immune responses. Specifically, RT + ATMi led to a reduction in the secretion of IL-8, IL-6, TNF-α, IL-13, IL-1β, and IL-10 in two or more HNSCC cell lines compared to RT alone. In contrast, the combined treatment of RT and ATRi increased the levels of secreted IL-8, IL-6, TNF-α, IL-13, IL-12p70, IL-1β and IL-10 in two or more cell lines compared to RT alone or RT + ATMi. Our analysis did not reveal significant changes in the secretion of IL-2, IL-4, and IFN-γ between the different treatment approaches.

The role of these cytokines in immune-related pro- and anti-tumor response remains ambivalent, as described by Jou et al. (65). Type 1 immunity cytokines like IL-12 and TNF-α have been shown to initiate anti-tumor immune response exhibited by NK cells, CD8^+^ T cells and T helper 1 (Th1) cells. Contrary, type 2 immunity related cytokines such as IL-4 and IL-13 were believed to predominantly mediate pro-tumoral effects. However, the notion that type 1 and type 2 cytokines act as tumor inhibitors and promoters respectively was found inadequate for describing the TME, as these cytokines were observed to exert varying functions depending on the context (65). Additionally, it is crucial to differentiate the direct effects of the studied cytokines on tumor cells from their role in triggering an immune response.

IL-8 is a pro-inflammatory cytokine that plays a role in recruiting leukocytes during injury and inflammation. However, in the context of several cancer entities, IL-8 was shown to exhibit pro-tumoral characteristics by affecting cancer cells themselves and altering the tumor microenvironment (66, 67). Overall, elevated levels of IL-8 in cancer patients typically align with advanced tumor stage, higher grading, and increased tumor burden, and seem to be predictive for resistance to immune checkpoint inhibitors (68, 69). Specifically, IL-8 is implicated in the progression of HNSCC through the CXCR1/2-mediated NOD1/RIP2 signaling pathway (70). Additionally, Chen et al. recently found that IL-8 enhances the invasion of HNSCC cells through the activation of STAT3 signaling pathway (71).

Similarly, IL-6 secreted by HNSCC cells initiates the STAT3 signaling pathway, thereby reducing NK cell activity and function, and enhancing HNSCC malignancy in conjunction with IL-8 (72). Additionally, it has been shown that IL-6 promotes carcinogenesis, progression and metastasis in HNSCC (73). Overall, IL-6 is associated with cancer development and progression by suppressing T cell activity against tumors and inhibition of dendritic cell maturation, among other mechanism (74).

We found that combined treatment of RT + ATMi significantly reduced IL-8 levels in all four cell lines and IL-6 levels in both HPV-negative cell lines compared to RT treatment alone. In contrast, RT + ATRi increased IL-8 levels in two of four cell lines, independent of the HPV status, and IL-6 levels in all four cell lines compared to single RT treatment. Considering the tumor-promoting properties of these cytokines, the combination therapy of RT + ATMi seems to outperform RT + ATRi in this scenario, contrary to our previous findings. However, similar to the application of PD-1 blockade, strategies aimed at decreasing IL-6 secretion could complement the combined treatment of RT + ATRi. In this regard, Yang et al. recently demonstrated that the simultaneous blockade of IL-6 and C-C motif chemokine receptor 2 (CCR2) enhanced the anti-tumor activity of NK cells in HNSCC (75).

Similar trends were observed in the secretion of IL-1β by the HNSCC cells in our experiments. Combining RT + ATMi reduced IL-1β levels compared to RT alone in three of the four cell lines, whereas RT + ATRi increased IL-1β secretion compared to RT + ATMi in corresponding cell lines. IL-1β is an inflammatory cytokine known to upregulate various molecules involved in disease pathology, including cytokines, chemokines, adhesion molecules, acute phase proteins, and tissue remodeling enzymes (76). Additionally, it is known to play a role in the vascularization and metastasis of malignant tumors (77). In the context of HNSCC, IL-1β was shown to be involved in several oncogenic mechanisms, such as the differentiation of tumor-associated macrophages which are associated with a poor prognosis (76, 78). Considering the tumor-promoting nature of IL-1β, lower IL-1β levels induced by the combination therapy of RT + ATMi are preferable. However, the dual treatment of RT + ATRi resulted in comparable levels of IL-1β compared to RT alone, suggesting that the addition of ATRi to RT may not exacerbate the effects of IL-1β secretion.

Regarding cancer, the role of TNF-α is a double-edged sword. The pro-inflammatory cytokine that is mainly produced by macrophages but also other immune cells and non-immune cells such as endothelial cells exhibits both tumor-promoting and tumor-suppressing roles in the TME (79). On the one hand, research has demonstrated that TNF-α promotes tumor growth by enhancing proliferation, survival, and angiogenesis in cancer cells that are resistant to its cytotoxic effects (80). Specifically, Zhang et al. recently showed that TNF-α promotes tumor lymph angiogenesis in HNSCC (81). On the other hand, TNF-α is known to diminish tumor cell proliferation and initiate tumor regression, mainly by the induction of cancer cell death (79, 80). For instance, TNF-α induces both apoptotic and necrotic cell death in a variety of cell types (77), potentially promoting immune-related anti-tumor response. Additionally, Calcinotti et al. found that a TNF-α fusion protein enhanced the effectiveness of immunotherapy through the upregulation of leukocyte-endothelial cell adhesion molecules, the secretion of proinflammatory cytokines and the infiltration of tumor-specific effector CD8^+^ T cells in tumor-bearing mice (82). In various animal cancer models, TNF-α exhibits broad anti-tumor effects by stimulating the immune system, yet it also presents significant toxicity at the same time (83). In our experiments, RT + ATRi led to enhanced levels of secreted TNF-α compared to single RT treatment or RT + ATMi. Further *in vivo* experiments are needed to examine whether this could exhibit pro- or anti-tumoral effects.

Like TNF-α, IL-13 prohibits both pro- and anti-tumoral effector functions. The cytokine has been shown to directly promote tumor growth, metastasis, and escape from apoptosis for certain tumor entities through several mechanisms (84). However, elevated levels of IL-13 are associated with better overall survival compared to low IL-13 levels in colorectal cancer (85), and further studies revealed anti-tumor effects of IL-13 *in vivo*. Ma et al. found that IL-13 diminishes tumorigenicity, presumably through the recruitment of neutrophils and macrophages and the enhancement of innate antitumor immunity (86). Overall, IL-13 is heavily involved in the initiation of a type-2 immunity (84), whose role in anti-tumor immunity is currently being examined (65). In our experiments, combining RT + ATMi reduced IL-13 levels in both HPV-negative HNSCC cells lines compared to RT alone. In contrast, RT + ATRi tendentially increased the secretion of IL-13, potentially inducing an immune response.

Some of the cytokines we studied clearly display anti-tumor properties. IL-12 has been shown to act as potent anti-tumor mediator in a variety of preclinical models (87). It is an effector cytokine involved in the polarization of CD4^+^ T cells towards a Th1 response (88) that is crucial for the activation of both cytotoxic T and NK cells and tumor clearance. However, IL-12 has not only shown to actively induce anti-tumor immunity, but also to inhibit immune suppression by altering processes involved in the survival and proliferation of Treg cells (89, 90). Although the systemic administration of IL-12 can be toxic (89), McMichael et al. recently showed in a phase I/II trial that administrating patients with unresectable HNSCC with cetuximab and IL-12 was well-tolerated, with several individuals showing extended periods of progression-free survival (91). In our study, combining RT with ATRi led to increased levels of secreted IL-12p70 compared to RT alone or RT + ATMi in two of the four cell lines, potentially contributing to enhanced anti-tumor response by activation of both innate and adaptive immunity.

In contrast to previously discussed cytokines, IL-10 has traditionally been viewed as an anti-inflammatory cytokine (92, 93), leading to the presumption that it attenuates the immune response against cancer. However, recent research has revealed that IL-10 possesses both pro- and anti-tumor properties, increasing interest in its potential for cytokine-based cancer immunotherapies. Studies have shown that overexpression of IL-10 in human cancer models promotes tumor rejection, induces durable immunity, and enhances the cytotoxicity of CD8^+^ T cells (94). Interestingly, Guo et al. recently showed that an IL-10-Fc fusion protein led to increased expansion and effector function of terminally exhausted CD8^+^ TILs, thereby enhancing immune-related anti-tumor response (95). These findings suggest that elevated IL-10 levels may play a role in enhancing T cell-mediated anti-tumor responses. In our experiments, combining RT with ATMi resulted in decreased IL-10 levels in three of four cell lines compared to single RT treatment, while RT + ATRi tendentially increased IL-10 secretion. This suggests once more that RT + ATRi might augment anti-tumor immunity.

The role of the cytokinome in HNSCC remains ambiguous, as described by Denaro et al. (61). Within the HNSCC TME, immune and cancer cells interact in a complex network, and the composition of both immune and non-immune cells undergo changes as the disease progresses. Consequently, the cytokinome is a subject to constant change, with both pro- and anti-tumoral effects of cytokines potentially co-existing at various stages of cancer progression (58). However, certain cytokines within the TME might be associated with the prognosis of HNSCC patients. For instance, it has been shown that the TME of HPV-negative HNSCC compromises higher levels of IL-6 compared to HPV-positive HNSCC and that enhanced IL-6 expression predicts a poor prognosis (96). Since HPV-positive HNSCC exhibits better treatment response, altered composition of the cytokinome in HPV negative HNSCC might impact treatment effectiveness. Considering the increased significance of the TME in tumor biology research is nowadays not focusing on the cancer cell solely but on involving the TME as a possible target for combinatory regimes. Nonetheless, advanced preclinical models, such as 3D models using spheroids and organoids will be necessary to investigate the TME deeper and ultimately adequate immune-competent animal models will be unavoidable to get more insides on the consequences of any therapy setting on the TME.

Whereas both ATMi and ATRi have already been shown to work as radiosensitizer *in vitro* and *in vivo* for several cancer entities, we here showed that combining RT with smKI also alters the immunogenicity of HNSCC cells (97). In our study, we demonstrated that the combination of RT with ATRi or ATMi had distinct impacts on the expression of ICM expression and the cytokine secretion from treated HNSCC cells, suggesting a potential enhancement of immune-mediated anti-tumor responses. Particularly, the synergy between RT and ATRi may augment this response by upregulating immunostimulatory ICMs and increasing the release of anti-tumor mediating cytokines. Contrary, combining ATMi with RT might even suppress immune-stimulatory effects triggered by RT. Further *in vivo* experiments are needed to validate potential pro- and anti-inflammatory effects of the altered ICM expression and cytokine secretion after RT treatment combined with smKI.

### 4.4 Identification of different gene subsets according to inhibitor treatment of Cal-33

Particularly, the treatment of HPV-negative HNSCC remains a challenge. Hence, accelerating immune-related anti-tumor response may be one promising approach to improve clinical outcome and survival rates. Although we already have knowledge of many of the correlations that cause changes in tumor cells after RT and after treatment with ATMi or ATRi, we still lack a detailed understanding of many molecular processes. To better understand the effects and consequences of inhibiting ATM or ATR concomitant to RT we analyzed the HNSCC cell line Cal-33 on mRNA level (RNAseq). Previous findings based on our multiplex ELISA analysis of the secretome were confirmed on mRNA level, such as a significant down-regulation of IL-6 in RT + ATMi treated Cal-33 cells. Further, a down-regulation of several immune modulators such as CD137-L, Galectin-9, IL-1β and IL-1α was observed after RT + ATMi treatment. Interestingly, we also found a down-regulation of CD70 in RT + ATMi treated cells. This contrasts with our findings of ICM surface expression, which showed no differences of CD70 expression in Cal-33 after RT nor a combination with ATMi or ATRi.

In principle, however, a more immunosuppressive phenotype can be determined by the therapy of HNSCC cells with RT + ATMi. It is possible that crucial targets that are necessary for a sufficient anti-tumor immune response in patients are inhibited here. This could be an indication of the expanding application of ATR inhibitors in clinical trials. However, the analysis of DEGs does not only identify a gene set that shows down-regulation by RT + ATMi. A gene set could also be identified in which genes are only down- or up-regulated by RT. Most interesting is a sub-group in which we observe down-regulation by the combination of RT + ATRi. Whether these tumor-specific changes also have a functional influence on the immune system must be clarified by further investigations. Co-cultures with cells of the adaptive or innate immune response would offer the possibility to investigate the interaction between tumor cells and immune cells.

The RNAseq analysis provided further indications of the possible superiority of a combination therapy with ATRi. In addition to the immunomodulatory targets that we found to be significantly regulated in our data set, we also identified a mechanistically decisive target. The central protein of the RNA-sensing pathway RIG-1 is also down-regulated by the combination of RT with ATMi. In parallel to the cGAS/STING-regulated DNA-sensing signaling pathway, RIG-1 is able to detect free cytoplasmic RNA and trigger a corresponding immune response (27). Further mechanisms are discussed in the literature that also implicate RIG-1 as an important part of DNA detection (98) and consequently a trigger of immune responses. If the sensors for cytoplasmic nucleic acids, induced by irradiation and DDR inhibition, are downregulated by ATMi, this could explain the inhibited immune response observed with this combination. Conclusively, it is possible that ATMi inhibits essential signaling pathways that are not affected by ATRi, which avoids an adequate anti-tumor response.

Finally, we detected a promising target, that may play a role in the limited effectiveness of treatment with ATMi. Our RNAseq analysis showed that the combination of RT + ATMi leads to the significant up-regulation of the protein EGF like repeats and discoidin domains 3 (EDIL3), also known as Developmental Endothelial Locus 1 (Del-1). EDIL3 acts as an anti-inflammatory factor and immune modulator triggering „immune execution” (99). Tabasum and colleagues recently deciphered the supportive role of EDIL3 leading to immune evasion through CD8+ T-cell exclusion in melanoma (28). Interestingly, EDIL3 expression was found to be present in different tumor types, such as breast cancer. Here, it has been recognized as a biomarker for early disease detection (100, 101). However, there is still no comprehensive data on the function of EDIL3 in HNSCC. The role of EDIL3 in HNSCC should be further deciphered in the future.

## 5 Conclusion

In conclusion, our data indicate that monotherapy with either ATR or ATM inhibitor alone does not lead to extensive cell death, but this effect is increased when combined with hypo-fractionated RT. Further, the immune phenotype of cancer cells, not dying from combination therapy itself, is altered predominantly by RT + ATRi in an immune-stimulatory manner by the up-regulation of ICOS-L. This might be advantageous for HPV-negative patients, since the combination of RT + DDRi shows promising effects to re-sensitize tumor cells to RT and further induce an immune-stimulatory phenotype triggering an anti-tumor response. However, the analysis of secreted cytokines after treatment of HNSCC cell lines revealed an ambivalent influence of both inhibitors, as we observed the intensified secretion of IL-6 and IL-8 after RT + ATRi. These findings were confirmed by RNAseq analysis, which further highlighted the immune-suppressive nature of RT + ATMi. We detected the down-regulation of a central protein of cytoplasmatic sensing pathways of nucleic acids, RIG-1, and found one immune-suppressive target, EDIL3, strongly up-regulated by RT + ATMi. It needs to be further investigated (*in vitro*, ex vivo and *in vivo*) whether the addition of ATM inhibitors or ATR inhibitors to RT leads to a comprehensive immune-response, which might help to explain the different efficiency of DDR inhibitors in clinical trials. Optimized and newly established models such 3-dimentional spheroid or organoid models, as well as co-culture setting, including tumor but also immune cells, are needed to overcome these challenges.

## Data Availability

The raw data supporting the conclusions of this article will be made available by the authors, without undue reservation.

## References

[1] Johnson DE Burtness B Leemans CR Lui VWY Bauman JE Grandis JR. Head and neck squamous cell carcinoma. Nat Rev Dis Primers. (2020) 6. doi: 10.1038/S41572-020-00224-3 PMC7944998 33243986

[2] Barsouk A Aluru JS Rawla P Saginala K Barsouk A. Epidemiology, risk factors, and prevention of head and neck squamous cell carcinoma. Med Sci. (2023) 11:42. doi: 10.3390/medsci11020042 PMC10304137 37367741

[3] Ang KK Harris J Wheeler R Weber R Rosenthal DI Nguyen-Tân PF. Human papillomavirus and survival of patients with oropharyngeal cancer. N Engl J Med. (2010) 363:24. doi: 10.1056/NEJMOA0912217 20530316 PMC2943767

[4] Zhou C Parsons JL. The radiobiology of HPV-positive and HPV-negative head and neck squamous cell carcinoma. Expert Rev Mol Med. (2020) 22. doi: 10.1017/ERM.2020.4 PMC7754878 32611474

[5] Long Z Grandis JR Johnson DE. Emerging tyrosine kinase inhibitors for head and neck cancer. Expert Opin Emerg Drugs. (2022) 27:333. doi: 10.1080/14728214.2022.2125954 36131561 PMC9987561

[6] Pereira D Martins D Mendes F. Immunotherapy in head and neck cancer when, how, and why? Biomedicines. (2022) 10. doi: 10.3390/biomedicines10092151 PMC9495940 36140252

[7] Afshari K Sohal KS. Potential alternative therapeutic modalities for management head and neck squamous cell carcinoma: A review. Cancer Control. (2023) 30. doi: 10.1177/10732748231185003 PMC10278411 37328298

[8] Kimple RJ Smith MA Blitzer GC Torres AD Martin JA Yang RZ. Enhanced radiation sensitivity in HPV-positive head and neck cancer. Cancer Res. (2013) 73:4791–800. doi: 10.1158/0008-5472.CAN-13-0587 PMC3732540 23749640

[9] Long D Xu L Deng Z Guo D Zhang Y Liu Z. HPV16 E6 enhances the radiosensitivity in HPV-positive human head and neck squamous cell carcinoma by regulating the miR-27a-3p/SMG1 axis. Infect Agents Cancer. (2021) 16:56. doi: 10.1186/s13027-021-00397-w PMC8361787 34389030

[10] Weber AM Ryan AJ. ATM and ATR as therapeutic targets in cancer. Pharmacol Ther. (2015) 149:124–38. doi: 10.1016/j.pharmthera.2014.12.001 25512053

[11] Karukonda P Odhiambo D Mowery YM. Pharmacologic inhibition of ataxia telangiectasia and Rad3-related (ATR) in the treatment of head and neck squamous cell carcinoma. Mol Carcinog. (2022) 61:225–38. doi: 10.1002/mc.23384 PMC8799519 34964992

[12] Priya B Ravi S Kirubakaran S. Targeting ATM and ATR for cancer therapeutics: Inhibitors in clinic. Drug Discovery Today. (2023) 28:103662. doi: 10.1016/J.DRUDIS.2023.103662 37302542

[13] Pal A Kundu R. Human papillomavirus E6 and E7: the cervical cancer hallmarks and targets for therapy. Front Microbiol. (2020) 10:3116. doi: 10.3389/fmicb.2019.03116 32038557 PMC6985034

[14] Ghittoni R Accardi R Chiocca S Tommasino M. Role of human papillomaviruses in carcinogenesis. ecancermedicalscience. (2015) 9. doi: 10.3332/ecancer.2015.526 PMC4431404 25987895

[15] Pike KG Barlaam B Cadogan E Campbell A Chen Y Colclough N. The identification of potent, selective, and orally available inhibitors of ataxia telangiectasia mutated (ATM) kinase: the discovery of AZD0156 (8-{6-[3-(Dimethylamino)propoxy]pyridin-3-yl}-3-methyl-1-(tetrahydro-2 H-pyran-4-yl)-1,3-dihydro-2 H-imidazo[4,5- c]quinolin-2-one). J Med Chem. (2018) 61:3823–41. doi: 10.1021/ACS.JMEDCHEM.7B01896/SUPPL_FILE/JM7B01896_SI_003.CSV 29683659

[16] Riches LC Trinidad AG Hughes G Jones GN Hughes AM Thomason AG. Pharmacology of the ATM inhibitor AZD0156: Potentiation of irradiation and olaparib responses preclinically’. Mol Cancer Ther. (2020) 19:13–25. doi: 10.1158/1535-7163.MCT-18-1394/88396/AM/PHARMACOLOGY-OF-THE-ATM-INHIBITOR-AZD0156 31534013

[17] Scheper J Hildebrand LS Fauhaber EM Deloch L Gaipl US Symank J. Tumor-specific radiosensitizing effect of the ATM inhibitor AZD0156 in melanoma cells with low toxicity to healthy fibroblasts. Strahlentherapie Und Onkologie. (2023) 199:1128. doi: 10.1007/S00066-022-02009-X 36229655 PMC10673781

[18] Leszczynska KB Dobrynin G Leslie RE Ient J Boumelha AJ Senra JM. Preclinical testing of an Atr inhibitor demonstrates improved response to standard therapies for esophageal cancer. Radiotherapy Oncol. (2016) 121:232. doi: 10.1016/J.RADONC.2016.10.023 PMC5154234 27839769

[19] Liu C Wang X Qin W Tu J Li C Zhao W. Combining radiation and the ATR inhibitor berzosertib activates STING signaling and enhances immunotherapy via inhibiting SHP1 function in colorectal cancer. Cancer Commun. (2023) 43:435. doi: 10.1002/CAC2.12412 PMC10091106 36855844

[20] Search for: berzosertib | Card Results | ClinicalTrials.gov . Available online at: https://clinicaltrials.gov/search?cond=berzosertib (Accessed Dec. 22, 2023).

[21] Faulhaber EM Jost T Symank J Scheper J Bürkel F Fietkau R. Kinase inhibitors of dna-pk, atm and atr in combination with ionizing radiation can increase tumor cell death in hnscc cells while sparing normal tissue cells. Genes (Basel). (2021) 12. doi: 10.3390/GENES12060925/S1 PMC8235750 34204447

[22] Dobler C Jost T Hecht M Fietkau R Distel L. Senescence induction by combined ionizing radiation and DNA damage response inhibitors in head and neck squamous cell carcinoma cells. Cells. (2020) 9. doi: 10.3390/CELLS9092012 PMC7563880 32883016

[23] Tosi A Parisatto B Menegaldo A Spinato G Guido M Mistro AD. The immune microenvironment of HPV-positive and HPV-negative oropharyngeal squamous cell carcinoma: a multiparametric quantitative and spatial analysis unveils a rationale to target treatment-naïve tumors with immune checkpoint inhibitors. J Exp Clin Cancer Res. (2022) 41:279. doi: 10.1186/S13046-022-02481-4 36123711 PMC9487049

[24] Wimmer S Deloch L Hader M Derer A Grottker F Weissmann T. Hypofractionated radiotherapy upregulates several immune checkpoint molecules in head and neck squamous cell carcinoma cells independently of the HPV status while ICOS-L is upregulated only on HPV-positive cells. Int J Mol Sci. (2021) 22. doi: 10.3390/IJMS22179114 PMC8430967 34502022

[25] Rujas E Cui H Sicard T Semesi A Julien JP. Structural characterization of the ICOS/ICOS-L immune complex reveals high molecular mimicry by therapeutic antibodies. Nat Commun. (2020) 11. doi: 10.1038/s41467-020-18828-4 PMC7545189 33033255

[26] Grottker F Gehre S Reichardt CM Sengedorji A Jost T Rieckmann T. Radiotherapy combined with docetaxel alters the immune phenotype of HNSCC cells and results in increased surface expression of CD137 and release of HMGB1 of specifically HPV-positive tumor cells. Neoplasia. (2023) 45:1476–5586. doi: 10.1016/J.NEO.2023.100944 PMC10589749 37857049

[27] Xu Xx Wan H Nie L Shao T xin Xiang L Shao Jz. RIG-I: a multifunctional protein beyond a pattern recognition receptor. Protein Cell. (2018) 9:246–53. doi: 10.1007/s13238-017-0431-5 PMC5829270 28593618

[28] Tabasum S Thapa D Giobbie-Hurder A Weirather JL Campisi M Schol PJ. EDIL3 as an angiogenic target of immune exclusion following checkpoint blockade. Cancer Immunol Res. (2023) 11:1493–507. doi: 10.1158/2326-6066.CIR-23-0171 PMC10618652 37728484

[29] Hader M Savcigil DP Rosin A Ponfick P Gekle S Wadepohl M. Differences of the immune phenotype of breast cancer cells after ex vivo hyperthermia by warm-water or microwave radiation in a closed-loop system alone or in combination with radiotherapy. Cancers (Basel). (2020) 12. doi: 10.3390/cancers12051082 PMC7281749 32349284

[30] Chen W Chen X Wang L Yang R Zhang W Zhang S. TIPE3 represses head and neck squamous cell carcinoma progression via triggering PGAM5 mediated mitochondria dysfunction. Cell Death Dis. (2023) 14:251. doi: 10.1038/s41419-023-05775-3 37024453 PMC10079926

[31] Li H Wawrose JS Gooding WE Garraway LA Lui VWY Peyser ND. Genomic analysis of head and neck squamous cell carcinoma cell lines and human tumors: a rational approach to preclinical model selection. Mol Cancer Res. (2014) 12:571–82. doi: 10.1158/1541-7786.MCR-13-0396 PMC3989421 24425785

[32] Poon IKH Lucas CD Rossi AG Ravichandran KS. Apoptotic cell clearance: Basic biology and therapeutic potential. Nat Rev Immunol. (2014) 14:166–80. doi: 10.1038/nri3607 PMC4040260 24481336

[33] Medina CB Mehrotra P Arandjelovic S Perry JSA Guo Y Morioka S. Metabolites released from apoptotic cells act as tissue messengers. Nature. (2020) 580:130–5. doi: 10.1038/s41586-020-2121-3 PMC7217709 32238926

[34] Frey B Rückert M Deloch L Rühle PF Derer A Fietkau R. Immunomodulation by ionizing radiation—impact for design of radio-immunotherapies and for treatment of inflammatory diseases. Immunol Rev. (2017) 280:231–48. doi: 10.1111/imr.12572 29027224

[35] Kroemer G Galassi C Zitvogel L Galluzzi L. Immunogenic cell stress and death. Nat Immunol. (2022) 23:487–500. doi: 10.1038/s41590-022-01132-2 35145297

[36] Schnoell J Sparr C Al-Gboore S Haas M Brkic FF Kadletz-Wanke L. The ATR inhibitor berzosertib acts as a radio- and chemosensitizer in head and neck squamous cell carcinoma cell lines. Invest New Drugs. (2023) 41:842. doi: 10.1007/S10637-023-01408-W 37934325 PMC10663216

[37] Gamrekelashvili J Greten TF Korangy F. Immunogenicity of necrotic cell death. Cell Mol Life Sci. (2018) 72:273–83. doi: 10.1007/s00018-014-1741-x.Immunogenicity PMC6309818 25274062

[38] Bertheloot D Latz E Franklin BS. Necroptosis, pyroptosis and apoptosis: an intricate game of cell death. Cell Mol Immunol. (2021) 18:1106–21. doi: 10.1038/s41423-020-00630-3 PMC8008022 33785842

[39] Kepp O Senovilla L Vitale I Vacchelli E Adjemian S Agostinis P. Consensus guidelines for the detection of immunogenic cell death. OncoImmunology. (2014) 3. doi: 10.4161/21624011.2014.955691 PMC4292729 25941621

[40] Krysko DV Garg AD Kaczmarek A Krysko O Agostinis P Vandenabeele P. Immunogenic cell death and DAMPs in cancer therapy. Nat Rev Cancer. (2012) 12:860–75. doi: 10.1038/nrc3380 23151605

[41] Cramer JD Burtness B Ferris RL. Immunotherapy for head and neck cancer: Recent advances and future directions. Oral Oncol. (2019) 99. doi: 10.1016/j.oraloncology.2019.104460 PMC7749717 31683169

[42] Meliante PG Barbato C Zoccali F Ralli M Greco A de Vincentiis M. Programmed cell death-ligand 1 in head and neck squamous cell carcinoma: molecular insights, preclinical and clinical data, and therapies. Int J Mol Sci. (2022) 23. doi: 10.3390/ijms232315384 PMC9738355 36499710

[43] Ferris RL Blumenschein G Jr. Fayette J Guigay J Colevas AD Licitra L. Nivolumab for recurrent squamous-cell carcinoma of the head and neck. Br Dent J. (2016) 221:632. doi: 10.1038/sj.bdj.2016.860 27857112

[44] Harrington KJ Burtness B Greil R Soulières D Tahara M de Castro G Jr.. Pembrolizumab with or without chemotherapy in recurrent or metastatic head and neck squamous cell carcinoma: updated results of the phase III KEYNOTE-048 study. J Clin Oncol. (2022) 41:790–802. doi: 10.1200/JCO.21 36219809 PMC9902012

[45] Almangush A De Keukeleire S Rottey S Ferdinande L Vermassen T Leivo I. Tumor-infiltrating lymphocytes in head and neck cancer: ready for prime time? Cancers (Basel). (2022) 14. doi: 10.3390/cancers14061558 PMC8946626 35326709

[46] Mandal R Şenbabaoğlu Y Desrichard A Havel JJ Dalin MG Riaz N. The head and neck cancer immune landscape and its immunotherapeutic implications. JCI Insight. (2016) 1:1–18. doi: 10.1172/jci.insight.89829 PMC5070962 27777979

[47] Want MY Bashir Z Najar RA. T cell based immunotherapy for cancer: approaches and strategies. Vaccines (Basel). (2023) 11:1–19. doi: 10.3390/vaccines11040835 PMC10142387 37112747

[48] Stevanović S Draper LM Langahn MM Campbell TE Kwong ML Wunderlich JR. Complete regression of metastatic cervical cancer after treatment with human papillomavirus-targeted tumor-infiltrating T cells. J Clin Oncol. (2015) 33:1543–50. doi: 10.1200/JCO.2014.58.9093 PMC4417725 25823737

[49] Wolchok JD Chiarion-Sileni V Gonzalez R Rutkowski P Grob JJ Cowey CL. Overall survival with combined nivolumab and ipilimumab in advanced melanoma. N Engl J Med. (2017) 377:1345–56. doi: 10.1056/NEJMoa1709684.Overall PMC5706778 28889792

[50] Marin-Acevedo JA Dholaria B Soyano AE Knutson KL Chumsri S Lou Y. Next generation of immune checkpoint therapy in cancer: New developments and challenges. J Hematol Oncol. (2018) 11:1–20. doi: 10.1186/s13045-018-0582-8 29544515 PMC5856308

[51] Melero I Hervas-Stubbs S Glennie M Pardoll DM Chen L. Immunostimulatory monoclonal antibodies for cancer therapy. Nat Rev Cancer. (2007) 7:95–106. doi: 10.1038/nrc2051 17251916

[52] Goodwin RG Din WS Davis-Smith T Anderson DM Gimpel SD Sato TA. Molecular cloning of a ligand for the inducible T cell gene 4-1BB: a member of an emerging family of cytokines with homology to tumor necrosis factor. Eur J Immunol. (1993) 23:2631–41. doi: 10.1002/eji.1830231037 8405064

[53] Khushalani NI Ott PA Ferris RL Cascone T Schadendorf D Le DT. Final results of urelumab, an anti-CD137 agonist monoclonal antibody, in combination with cetuximab or nivolumab in patients with advanced solid tumors. J Immunother Cancer. (2024) 12. doi: 10.1136/jitc-2023-007364 PMC10921538 38458639

[54] Srivastava RM Trivedi S Concha-Benavente F Gibson SP Reeder C Ferrone S. CD137 stimulation enhances cetuximab induced natural killer (NK): dendritic cell (DC) priming of anti-tumor T cell immunity in head and neck cancer patients. Clin Cancer Res. (2018) 23:707–16. doi: 10.1158/1078-0432.CCR-16-0879.CD137 PMC5290200 27496866

[55] Lucido CT Vermeer PD Wieking BG Vermeer DW Lee JH. CD137 enhancement of HPV positive head and neck squamous cell carcinoma tumor clearance. Vaccines (Basel). (2014) 2:841–53. doi: 10.3390/vaccines2040841 PMC4429800 25984365

[56] Waldman AD Fritz JM Lenardo MJ. A guide to cancer immunotherapy: from T cell basic science to clinical practice. Nat Rev Immunol. (2020) 20:651–68. doi: 10.1038/s41577-020-0306-5 PMC7238960 32433532

[57] Burugu S Dancsok AR Nielsen TO. Emerging targets in cancer immunotherapy. Semin Cancer Biol. (2018) 52:39–52. doi: 10.1016/j.semcancer.2017.10.001 28987965

[58] Hutloff A Dittrich AM Beier KC Eljaschewitsch B Kraft R Anagnostopoulos I. ICOS is an inducible T-cellco-stimulator structurally andfunctionally related to CD28. Nature. (1999) 397:263–6. doi: 10.1038/16717 9930702

[59] Montes-Casado M Ojeda G Aragoneses-Fenoll L López D de Andrés B Gaspar ML. ICOS deficiency hampers the homeostasis, development and function of NK cells. PloS One. (2019) 14:1–26. doi: 10.1371/journal.pone.0219449 PMC6613708 31283790

[60] Hayman TJ Bhatia AK Jethwa KR Young MR Park HS. Combinations of immunotherapy and radiation therapy in head and neck squamous cell carcinoma: A narrative review. Transl Cancer Res. (2021) 10:2571–85. doi: 10.21037/tcr-20-2096 PMC8798834 35116571

[61] Denaro N Solinas C Garrone O Cauchi C Ruatta F Wekking D. The role of cytokinome in the HNSCC tumor microenvironment: A narrative review and our experience. Diagnostics. (2022) 12. doi: 10.3390/diagnostics12112880 PMC9689412 36428939

[62] Denaro N Merlano MC Lo Nigro C. Further understanding of the immune microenvironment in head and neck squamous cell carcinoma: Implications for prognosis. Cancer Manage Res. (2021) 13:3973–80. doi: 10.2147/CMAR.S277907 PMC8139676 34040438

[63] Economopoulou P Kotsantis I Psyrri A. Tumor microenvironment and immunotherapy response in head and neck cancer. Cancers. (2020) 12:1–23. doi: 10.3390/cancers12113377 PMC7696050 33203092

[64] Kartikasari AER Huertas CS Mitchell A Plebanski M. Tumor-induced inflammatory cytokines and the emerging diagnostic devices for cancer detection and prognosis. Front Oncol. (2021) 11:692142. doi: 10.3389/fonc.2021.692142 34307156 PMC8294036

[65] Jou E. Type 1 and type 2 cytokine-mediated immune orchestration in the tumour microenvironment and their therapeutic potential. Explor Targeted Anti-tumor Ther. (2023) 4:474–97. doi: 10.37349/etat.2023.00146 PMC10345208 37455828

[66] Fousek K Horn LA Palena C. Interleukin-8: A chemokine at the intersection of cancer plasticity, angiogenesis, and immune suppression. Pharmacol Ther. (2021) 219. doi: 10.1016/j.pharmthera.2020.107692 PMC8344087 32980444

[67] David JM Dominguez C Hamilton DH Palena C. The IL-8/IL-8R axis: A double agent in tumor immune resistance. Vaccines. (2016) 4. doi: 10.3390/vaccines4030022 PMC5041016 27348007

[68] Bakouny Z Choueiri TK. IL-8 and cancer prognosis on immunotherapy. Nat Med. (2020) 26:650–1. doi: 10.1038/s41591-020-0873-9 32405061

[69] Yuen KC Liu LF Gupta V Madireddi S Keerthivasan S Li C. High systemic and tumor-associated IL-8 correlates with reduced clinical benefit of PD-L1 blockade. Nat Med. (2020) 26:693–8. doi: 10.1038/s41591-020-0860-1 PMC8286544 32405063

[70] Chan L-P Wang L-F Chiang F-Y Lee K-W Kuo P-L Liang C-H. ‘IL-8 promotes HNSCC progression on CXCR1/2-meidated NOD1/RIP2 signaling pathway. ’ Oncotarget (2016) 7(38):61820–31. doi: 10.18632/oncotarget.11445 PMC5308693 27557518

[71] Chen Y Huang L Gan RH Yuan S Lan T Zheng D. IL-8 activates fibroblasts to promote the invasion of HNSCC cells via STAT3-MMP1. Cell Death Discovery. (2024) 10. doi: 10.1038/s41420-024-01833-7 PMC10847094 38320998

[72] Wu J Gao FX Wang C Qin M Han F Xu T. IL-6 and IL-8 secreted by tumour cells impair the function of NK cells via the STAT3 pathway in oesophageal squamous cell carcinoma. J Exp Clin Cancer Res. (2019) 38. doi: 10.1186/s13046-019-1310-0 PMC6642486 31324197

[73] Uz U Eskiizmir G. Association between interleukin-6 and head and neck squamous cell carcinoma: A systematic review. Clin Exp Otorhinolaryngology. (2021) 14:50–60. doi: 10.21053/ceo.2019.00906 PMC7904429 33587847

[74] Hirano T. IL-6 in inflammation, autoimmunity and cancer. Int Immunol. (2021) 33:127–48. doi: 10.1093/intimm/dxaa078 PMC7799025 33337480

[75] Yang F Yuan C Chen F Qin ZS Schmitt NC Lesinski GB. Combined IL6 and CCR2 blockade potentiates antitumor activity of NK cells in HPV-negative head and neck cancer. J Exp Clin Cancer Res. (2024) 43. doi: 10.1186/s13046-024-03002-1 PMC10929116 38468260

[76] Niklander SE Murdoch C Hunter KD. IL-1/IL-1R signaling in head and neck cancer. Front Oral Health. (2021) 2:722676. doi: 10.3389/froh.2021.722676 35048046 PMC8757896

[77] Voronov E Shouval DS Krelin Y Cagnano E Benharroch D Iwakura Y. IL-1 is required for tumor invasiveness and angiogenesis. Proc Natl Acad Sci U S A. (2003) 100(5):2645–50. Available online at: www.pnas.orgcgidoi10.1073pnas.0437939100. 10.1073/pnas.0437939100 PMC151394 12598651

[78] He KF Zhang L Huang CF Ma SR Wang YF Wang WM. CD163+ tumor-associated macrophages correlated with poor prognosis and cancer stem cells in oral squamous cell carcinoma. BioMed Res Int. (2014) 2014. doi: 10.1155/2014/838632 PMC4032721 24883329

[79] Laha D Grant R Mishra P Nilubol N. The role of tumor necrosis factor in manipulating the immunological response of tumor microenvironment. Front Immunol. (2021) 12:656908. doi: 10.3389/fimmu.2021.656908 33986746 PMC8110933

[80] Wang X Lin Y. ‘Tumor necrosis factor and cancer, buddies or foes? ’ Acta Pharmacol Sin (2008) 29:1275–88. doi: 10.1111/j.1745-7254.2008.00889.x PMC2631033 18954521

[81] Zhang C Zhu M Wang W Chen D Chen S Zheng H. TNF-α promotes tumor lymph angiogenesis in head and neck squamous cell carcinoma through regulation of ERK3. Transl Cancer Res. (2019) 8:2439–48. doi: 10.21037/tcr.2019.09.60 PMC8798551 35116996

[82] Calcinotto A Grioni M Jachetti E Curnis F Mondino A Parmiani G. Targeting TNF-α to neoangiogenic vessels enhances lymphocyte infiltration in tumors and increases the therapeutic potential of immunotherapy. J Immunol. (2012) 188:2687–94. doi: 10.4049/jimmunol.1101877 22323546

[83] Shen J Xiao Z Zhao Q Li M Wu X Zhang L. Anti-cancer therapy with TNFα and IFNγ: A comprehensive review. Cell Proliferation. (2018) 51. doi: 10.1111/cpr.12441 PMC6528874 29484738

[84] Terabe M Park JM Berzofsky JA. Role of IL-13 in regulation of anti-tumor immunity and tumor growth. Cancer Immunology Immunotherapy. (2004), 79–85. doi: 10.1007/s00262-003-0445-0 14610620 PMC11034335

[85] Formentini A Braun P Fricke H Link KH Henne-Bruns D Kornmann M. Expression of interleukin-4 and interleukin-13 and their receptors in colorectal cancer. Int J Colorectal Dis. (2012) 27:1369–76. doi: 10.1007/s00384-012-1456-0 22441356

[86] Ma HL Whitters MJ Jacobson BA Donaldson DD Collins M Dunussi-Joannopoulos K. Tumor cells secreting IL-13 but not IL-13Rα2 fusion protein have reduced tumorigenicity in *vivo* . Int Immunol. (2004) 16:1009–17. doi: 10.1093/intimm/dxh105 15184346

[87] Tugues S Burkhard SH Ohs I Vrohlings M Nussbaum K Vom Berg J. New insights into IL-12-mediated tumor suppression. Cell Death Differentiation. (2015) 22:237–46. doi: 10.1038/cdd.2014.134 PMC4291488 25190142

[88] Ylikoski E Lund R Kyläniemi M Filén S Kilpeläinen M Savolainen J. IL-12 up-regulates T-bet independently of IFN-γ in human CD4+ T cells. Eur J Immunol. (2005) 35:3297–306. doi: 10.1002/eji.200526101 16220539

[89] Mirlekar B Pylayeva-Gupta Y. IL-12 family cytokines in cancer and immunotherapy. Cancers. (2021) 13:1–23. doi: 10.3390/cancers13020167 PMC7825035 33418929

[90] Cao X Leonard K Collins LI Cai SF Mayer JC Payton JE. Interleukin 12 stimulates IFN-γ-mediated inhibition of tumor-induced regulatory T-cell proliferation and enhances tumor clearance. Cancer Res. (2009) 69:8700–9. doi: 10.1158/0008-5472.CAN-09-1145 PMC2783758 19843867

[91] McMichael EL Benner B Atwal LS Courtney NB Mo X Davis ME. A phase I/II trial of cetuximab in combination with interleukin-12 administered to patients with unresectable primary or recurrent head and neck squamous cell carcinoma. Clin Cancer Res. (2019) 25:4955–65. doi: 10.1158/1078-0432.CCR-18-2108 PMC6697573 31142501

[92] O’Garra A Barrat FJ Castro AG Vicari A Hawrylowicz C. Strategies for use of IL-10 or its antagonists in human disease. Immunol Rev. (2008) 223:114–31. doi: 10.1111/j.1600-065X.2008.00635.x 18613832

[93] Fiorentino DF Bond MW Mosmann TR. Two types of mouse T helper cell. IV. Th2 clones secrete a factor that inhibits cytokine production by Th1 clones. J Exp Med. (1989) 170(6):2081–95. doi: 10.1084/jem.170.6.2081 PMC2189521 2531194

[94] Oft M. IL-10: master switch from tumor-promoting inflammation to antitumor immunity. Cancer Immunol Res. (2014) 2:194–9. doi: 10.1158/2326-6066.CIR-13-0214 24778315

[95] Guo Y Xie YQ Gao M Zhao Y Franco F Wenes M. Metabolic reprogramming of terminally exhausted CD8+ T cells by IL-10 enhances anti-tumor immunity. Nat Immunol. (2021) 22:746–56. doi: 10.1038/s41590-021-00940-2 PMC7610876 34031618

[96] Gao J Zhao S Halstensen TS. Increased interleukin-6 expression is associated with poor prognosis and acquired cisplatin resistance in head and neck squamous cell carcinoma. Oncol Rep. (2016) 35:3265–74. doi: 10.3892/or.2016.4765 PMC4869939 27108527

[97] Classen S Petersen C Borgmann K. Crosstalk between immune checkpoint and DNA damage response inhibitors for radiosensitization of tumors. Strahlenther Onkol. (2023) 199:1152–63. doi: 10.1007/s00066-023-02103-8 PMC10674014 37420037

[98] Zhao Y Karijolich J. Know thyself: RIG-I-like receptor sensing of DNA virus infection. J Virol. (2019) 93. doi: 10.1128/jvi.01085-19 PMC6854496 31511389

[99] Gasca J Flores ML Jiménez-Guerrero R Sáez ME Barragán I Ruíz-Borrego M. EDIL3 promotes epithelial–mesenchymal transition and paclitaxel resistance through its interaction with integrin αVβ3 in cancer cells. Cell Death Discovery. (2020) 6. doi: 10.1038/s41420-020-00322-x PMC7494865 33014430

[100] Moon PG Lee JE Cho YE Lee SJ Jung JH Chae YS. Identification of developmental endothelial locus-1 on circulating extracellular vesicles as a novel biomarker for early breast cancer detection. Clin Cancer Res. (2016) 22:1757–66. doi: 10.1158/1078-0432.CCR-15-0654 26603257

[101] Lee JE Moon PG Cho YE Kim YB Kim IS Park H. Identification of EDIL3 on extracellular vesicles involved in breast cancer cell invasion. J Proteomics. (2016) 131:17–28. doi: 10.1016/j.jprot.2015.10.005 26463135

