# Corrigendum: Inhibition of ATM or ATR in combination with hypo-fractionated radiotherapy leads to a different immunophenotype on transcript and protein level in HNSCC

**DOI:** 10.3389/fonc.2025.1607882

**Published:** 2025-04-23

**Authors:** Julia Meidenbauer, Matthias Wachter, Sebastian R. Schulz, Nada Mostafa, Lilli Zülch, Benjamin Frey, Rainer Fietkau, Udo S. Gaipl, Tina Jost

**Affiliations:** ^1^ Translational Radiobiology, Department of Radiation Oncology, Uniklinikum Erlangen, Friedrich-Alexander-Universität Erlangen-Nürnberg, Erlangen, Germany; ^2^ Department of Radiation Oncology, Uniklinikum Erlangen, Friedrich-Alexander-Universität Erlangen-Nürnberg, Erlangen, Germany; ^3^ Comprehensive Cancer Center Erlangen-Europäische Metropolregion Nürnberg (EMN), Uniklinikum Erlangen, Friedrich-Alexander-Universität Erlangen-Nürnberg, Erlangen, Germany; ^4^ Division of Molecular Immunology, Internal Medicine III, University Hospital Erlangen, Nikolaus-Fiebiger Center, Friedrich-Alexander-University Erlangen-Nürnberg, Erlangen, Germany; ^5^ Deutsches Zentrum Immuntherapie, Uniklinikum Erlangen, Erlangen, Germany; ^6^ FAU Profile Center Immunomedicine Friedrich-Alexander-Universität Erlangen-Nürnberg, Erlangen, Germany

**Keywords:** HNSCC, DNA damage repair, kinase inhibitors, immunomodulation, ATM inhibition, ATR inhibition

In the published article, there was an error in the **Funding** statement. In the Funding statement one sentence was missing. The correct **Funding** statement appears below.

“The author(s) declare that financial support was received for the research, authorship, and/or publication of this article. This work was supported by the DFG Doctoral Training Group 2599 “Fine-Tuners of the Adaptive Immune Response” and partly by the Interdisciplinary Center for Clinical Research (IZKF) in Erlangen. The present work was performed by Julia Meidenbauer in (partial) fulfilment of the requirements for obtaining the degree “Dr. med.” at the Friedrich-Alexander-Universität Erlangen-Nürnberg (FAU).”

The authors apologize for this error and state that this does not change the scientific conclusions of the article in any way. The original article has been updated.

